# Enhanced potency of an IgM-like nanobody targeting conserved epitope in SARS-CoV-2 spike N-terminal domain

**DOI:** 10.1038/s41392-024-01847-8

**Published:** 2024-05-13

**Authors:** Bo Liu, Honghui Liu, Pu Han, Xiaoyun Wang, Chunmei Wang, Xinxin Yan, Wenwen Lei, Ke Xu, Jianjie Zhou, Jianxun Qi, Ruiwen Fan, Guizhen Wu, Wen-xia Tian, George F. Gao, Qihui Wang

**Affiliations:** 1 College of Veterinary Medicine, Shanxi Agricultural University, 030801 Jinzhong, China; 2 CAS Key Laboratory of Pathogen Microbiology and Immunology, Institute of Microbiology, Chinese Academy of Sciences (CAS), 100101 Beijing, China; 3 School of Life Sciences, Yunnan University, 650504 Kunming, Yunnan Province China; 4 NHC Key Laboratory of Biosafety, National Institute for Viral Disease Control and Prevention, Chinese Center for Disease Control and Prevention (China CDC), 102206 Beijing, China; 5 Savaid Medical School, University of Chinese Academy of Sciences, 101408 Beijing, China

**Keywords:** Infectious diseases, Immunotherapy

## Abstract

Almost all the neutralizing antibodies targeting the receptor-binding domain (RBD) of spike (S) protein show weakened or lost efficacy against severe acute respiratory syndrome coronavirus 2 (SARS-CoV-2) emerged or emerging variants, such as Omicron and its sub-variants. This suggests that highly conserved epitopes are crucial for the development of neutralizing antibodies. Here, we present one nanobody, N235, displaying broad neutralization against the SARS-CoV-2 prototype and multiple variants, including the newly emerged Omicron and its sub-variants. Cryo-electron microscopy demonstrates N235 binds a novel, conserved, cryptic epitope in the N-terminal domain (NTD) of the S protein, which interferes with the RBD in the neighboring S protein. The neutralization mechanism interpreted via flow cytometry and Western blot shows that N235 appears to induce the S1 subunit shedding from the trimeric S complex. Furthermore, a nano-IgM construct (MN235), engineered by fusing N235 with the human IgM Fc region, displays prevention via inducing S1 shedding and cross-linking virus particles. Compared to N235, MN235 exhibits varied enhancement in neutralization against pseudotyped and authentic viruses in vitro. The intranasal administration of MN235 in low doses can effectively prevent the infection of Omicron sub-variant BA.1 and XBB in vivo, suggesting that it can be developed as a promising prophylactic antibody to cope with the ongoing and future infection.

## Introduction

The highly contagious Omicron and its sub-variants of severe acute respiratory syndrome coronavirus 2 (SARS-CoV-2) have been driving a global surge of infections. In March 2023, the World Health Organization (WHO) updated its tracking system and working definitions for SARS-CoV-2 variants. Omicron sub-lineages have been considered independently as variants under monitoring (VUMs), variants of concern (VOCs) and variants of interest (VOIs) (https://www.who.int/). The sub-variants XBB.1.5, XBB.1.16, and EG.5 have maintained their dominance in several countries over the past year (https://ngdc.cncb.ac.cn/ncov/).^1^ Furthermore, the recent emergence of Omicron sub-variant BA.2.86, marked by more than 30 additional mutations in its spike (S) protein compared to sub-variant XBB.1.5, highlights ongoing viral evolution and raises concerns about potential immune evasion. Subsequently, BA.2.86 rapidly evolved into JN.1, which quickly became the predominant variant globally. While currently approved vaccines are effective in preventing COVID-19, particularly severe diseases, significant challenges persist in controlling both the current pandemic and potential future infections. For example, if individuals with immunodeficiencies, pregnancy, or a severe allergy to the vaccine’s components are not recommended to receive vaccines to stimulate active immunity, they may benefit from passive immunity via neutralizing antibodies.

Neutralizing antibodies with high affinity and specificity are safe and effective strategies for preventing and treating infections. The S1 subunit of the S protein is a major target for neutralizing antibodies, which is composed of a receptor-binding domain (RBD) and an N-terminal domain (NTD). Unlike RBD, which is responsible for binding to angiotensin-converting enzyme 2 (ACE2)^2,3^ receptor, the function of NTD is still not well understood. The neutralizing antibodies targeting RBD exert antiviral effects primarily by blocking the RBD binding to human ACE2^4–6^ and have previously been developed as a priority. However, the epitopes in the RBD are susceptible to immune escape and mutations, resulting in antibody resistance.^7–9^ Although BA.2.86 does not demonstrate more resistance to human sera than XBB.1.5 and EG.5.1,^10^ its descendant variant JN.1 continues to evolve and demonstrates a stronger immune evasion to monoclonal antibodies (mAbs) targeting RBD.^11^ Furthermore, the previously-authorized mAbs targeting RBD have demonstrated reduced or lost efficacy against the Omicron and its sub-variants, causing their emergency use authorizations to be revoked (https://www.fda.gov/). Thus, antibodies targeting non-RBD epitopes require attention. Recent studies suggested that NTD may be involved in viral entry and infection via regulating conformational change of the S^12,13^ or binding to glycans on the cell surface,^14^ thus it also could be developed as a target to induce epitope-specific antibodies.

Multiple NTD-directed neutralizing antibodies have been reported^15–17^ and are categorized into three groups (NTD-1, NTD-2, and NTD-3) based on their epitopes.^18^ Within the NTD-1 group, 4A8^15^ recognized the antigenic supersite to neutralize viruses. NTD-2 group antibody CV3-13^19^ approached the NTD at a nearly perpendicular angle relative to the S trimer axis. NTD-3 group antibody PVI.V6-14^16^ inserted its heavy chain complementarity-determining region 3 (HCDR3) loop into a hydrophobic cavity that was previously shown to bind a heme metabolite, biliverdin.^20^ S2L20^17,21^ and C1717^22^ are two antibodies targeting undefined epitopes, with S2L20 binding to an epitope flanked by glycans at positions N17, N61, and N234, and C1717 recognizing the viral membrane proximal side of the NTD and SD2 domain. NTD-targeting antibodies exhibit diverse epitopes and varying responses to Omicron sub-variants due to their distinct mutations on NTD. However, certain neutralizations were severely impaired by mutations within the NTD. For instance, the V213G mutation present in multiple Omicron sub-variants could lead to extensive evasion of neutralizing antibodies targeting NTD.^23^ Despite the hydrophobic cavity domain in the NTD is considered as a promising target for developing neutralizing antibodies against Omicron, it is reported to be susceptible to immune escape, particularly against BA.2.^24^ Additionally, no one targeting NTD is licensed for clinical trials yet (https://clinicaltrials.gov/). Nevertheless, it is essential to comprehensively map the epitope landscape of the NTD and explore the conserved neutralization epitopes within it as promising candidates for developing cross-reactive antibodies against continuously evolving variants.

Here, we selected multiple nanobodies targeting the NTD and focused on N235 that conferred high neutralization and good resistance to mutations in the current Omicron sub-variants as well as previously-circulating SARS-CoV-2 variants, including VOCs Alpha, Beta, Gamma and Delta. Furthermore, the nano-IgM antibody (MN235), engineered by fusing N235 with the human IgM Fc region, displayed remarkable improvement in neutralizing both pseudotyped and authentic viruses in vitro and prophylactic protective efficacy in vivo. The cryogenic electron microscopy (cryo-EM) structure showed that N235, which engages a highly conserved distinct cryptic epitope in the NTD, appears to exert a neutralizing effect by interfering with the neighboring RBD and inducing the S1 subunit shedding from the trimeric S complex. Altogether, our work demonstrates that nanobody N235 targets conserved distinct epitopes in the NTD, and its engineered decameric IgM form can be developed as a promising antibody-based therapy to address the ongoing and future pandemic.

## Results

### Nanobodies targeting the SARS-CoV-2 S NTD present cross-reactive activity

Two alpacas were sequentially immunized subcutaneously with SARS-CoV-2 prototype (PT) S and NTD proteins, five and four times, respectively. The NTD-specific nanobodies were generated using phage-display technology, along with phage-ELISA and flow cytometry assay (Supplementary Fig. 1a). The positive clones in phage-ELISA assay were subjected to sequencing and alignment analysis. The amino acid composition was determined for 35 nanobodies with different complementarity-determining regions (CDRs), 21 of which displayed binding to S proteins expressed on cells (Supplementary Fig. 1b and c). Nine of the 21 showed neutralization activity against the pseudotyped virus of the SARS-CoV-2 PT, with half-maximal inhibitory concentration (IC_50_) values ranging from 2.6 to 76.0 μg/mL (Supplementary Fig. 1d).

**Fig. 1:**
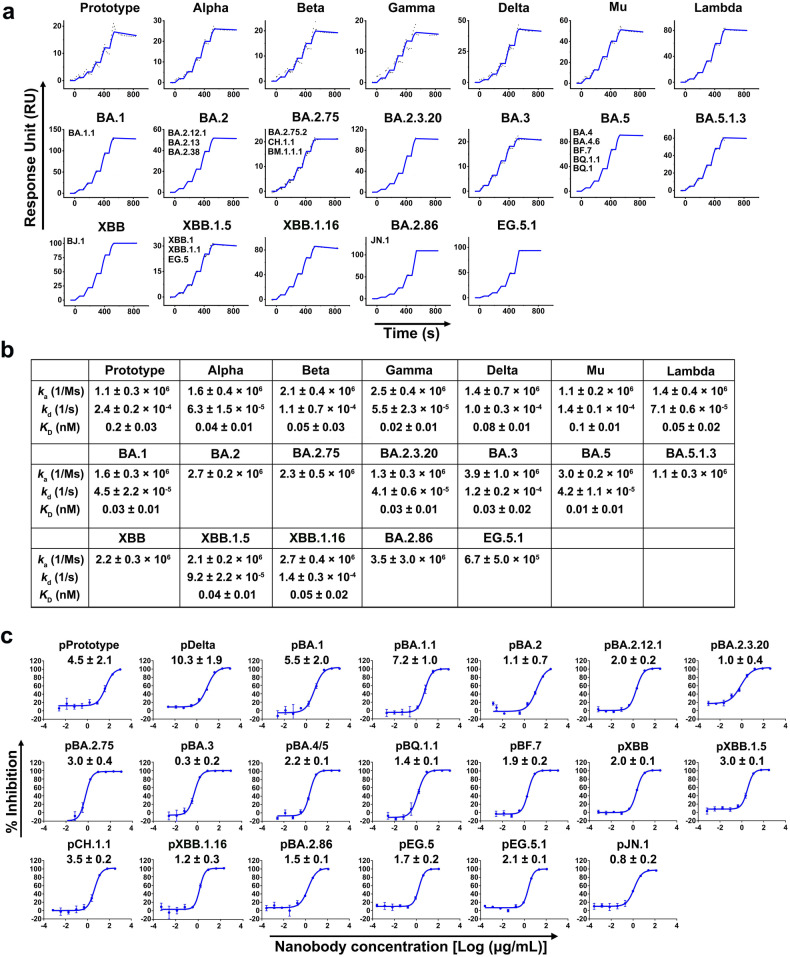
Characterization of cross-reactive nanobody N235 to multiple variants. **a** The binding kinetics of N235 to the NTDs from multiple variants were obtained using a Biacore 8 K system in single-cycle mode. Omicron sub-variants with identical NTD amino acid sequences are represented by the same binding curves. **b** Kinetic and affinity values (*k*_a_, *k*_d_, and *K*_D_) are the mean ± standard deviation (s.d.) of three independent results. The absence of annotated *k*_d_ and *K*_D_ represents no detectable dissociation from the NTDs. The dashed line underneath represents the raw data, and the kinetic fit is shown as a solid line. **c** Neutralization of pseudotyped variants by N235 in vero cells in vitro; *n* = 3 per dilution. Experiments were repeated independently three times with similar results and one representative curve is displayed. The IC_50_ values (μg/mL) are presented as the mean ± s.d. of three independent results

Omicron has developed into multiple sub-variants, causing global concern due to substantial mutations allowing immune escape. Therefore, we evaluated the efficacy of NTD-directed nanobodies against Omicron and its sub-variants. One nanobody named N235 had distinct CDRs (Supplementary Fig. 2) from the 21 nanobodies and exhibited high-affinity binding (*K*_D_ < 0.2 nM) to multiple strains, including PT, Alpha, Beta, Gamma, Delta, Mu, Lambda and Omicron sub-variants (BA.1, BA.2, BA.2.75, BA.2.3.20, BA.3, BA.5, BA.5.1.3, XBB, XBB.1.5, XBB.1.16, EG.5.1, BA.2.86 and JN.1) (Fig. 1a and b), indicating its cross-reactivity and potent neutralization potential. Additionally, N235 showed cross-neutralizing against multiple strains, including PT, Delta and multiple Omicron sub-variants (BA.1, BA.1.1, BA.2, BA.2.12.1, BA.2.3.20, BA.2.75, BA.3, BA.4/5, BQ.1.1, BF.7, XBB, XBB.1.5, XBB.1.16, CH.1.1, EG.5, EG.5.1, BA.2.86 and JN.1) (Fig. 1c). These results demonstrated that N235 may recognize a potentially highly conserved neutralizing epitope in the NTD of SARS-CoV-2.

### IgM-like antibody displays enhanced potency

**Fig. 2:**
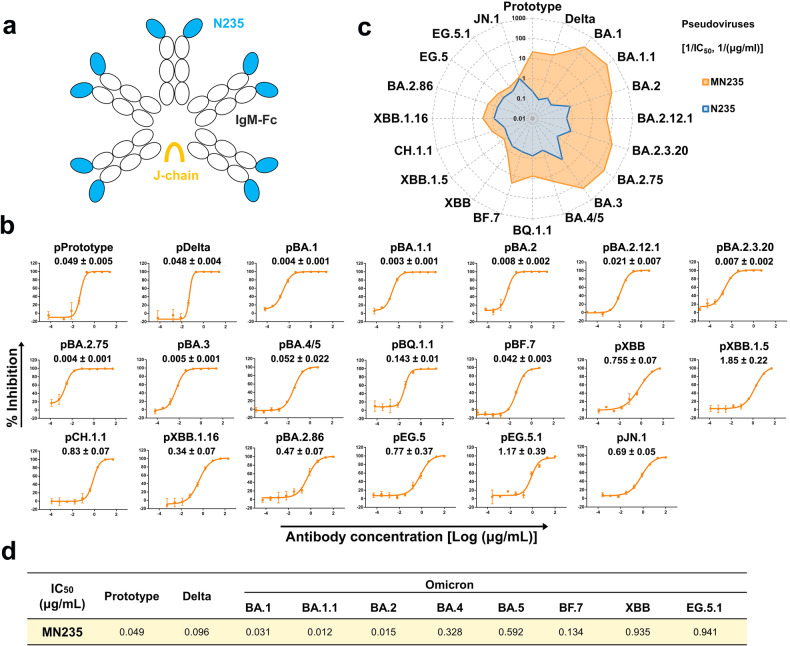
Engineering IgM-like MN235. **a** The IgM-like MN235 was constructed through nanobody N235 fused to the Fc of IgM. **b** Neutralization assay of pseudovirus in Vero cells. IC_50_ values for pseudoviruses are the mean ± s.d. of three independent results. Experiments were independently repeated three times with similar results, and one representative curve was displayed. **c** IC_50_ values (μg/mL) in pseudovirus-based neutralization assay are projected onto the radar chart, displaying N235 and MN235 in two dimensions. **d** IC_50_ values for authentic viruses are the mean of two independent experiments

Based on the practice of recombinant IgM-like antibody in our lab,^6^ we engineered decametric N235 (MN235) by fusing N235 with human IgM Fc to improve neutralizing efficacy (Fig. 2a). In the pseudovirus-based neutralization assay, we found that MN235 neutralized PT, Delta, and Omicron and its sub-variants (BA.1, BA.1.1, BA.2, BA.2.12.1, BA.2.75, BA.3, BA.4, BA.5, BQ.1.1 and BF.7), with comparable IC_50_ values of 0.003–0.143 μg/mL (Fig. 2b). This was more than 10-fold lower than the parental nanobody N235 (Fig. 2c). Notably, the neutralization capacity against BA.1 and BA.1.1 showed more than 1000-fold improvements compared to N235 (Fig. 2c). Moreover, MN235 neutralized pseudotyped Delta, BA.2, BA.2.3.20 and BA.2.75 exhibited 100-1,000-fold enhancement compared to N235 (Fig. 2c). Nevertheless, we found that MN235 neutralized Omicron sub-variants (XBB, XBB.1.5, XBB.1.16, CH.1.1, EG.5, EG.5.1, BA.2.86 and JN.1), with IC_50_ value of 0.34–1.85 μg/mL, which is comparable to that of N235. In addition, MN235 could also neutralize the authentic SARS-CoV-2 PT, Delta and Omicron sub-variants (BA.1, BA.1.1, BA.2, BA.4, BA.5, BF.7, XBB and EG.5.1), with IC_50_ values of 0.012–0.941 μg/mL (Fig. 2d). This finding is consistent with the pseudovirus-based neutralization results, where MN235 exhibited higher neutralization against BA.1, BA.1.1, and BA.2 compared to the other tested strains.

### N235 recognizes a conserved and cryptic epitope in NTD

**Fig. 3:**
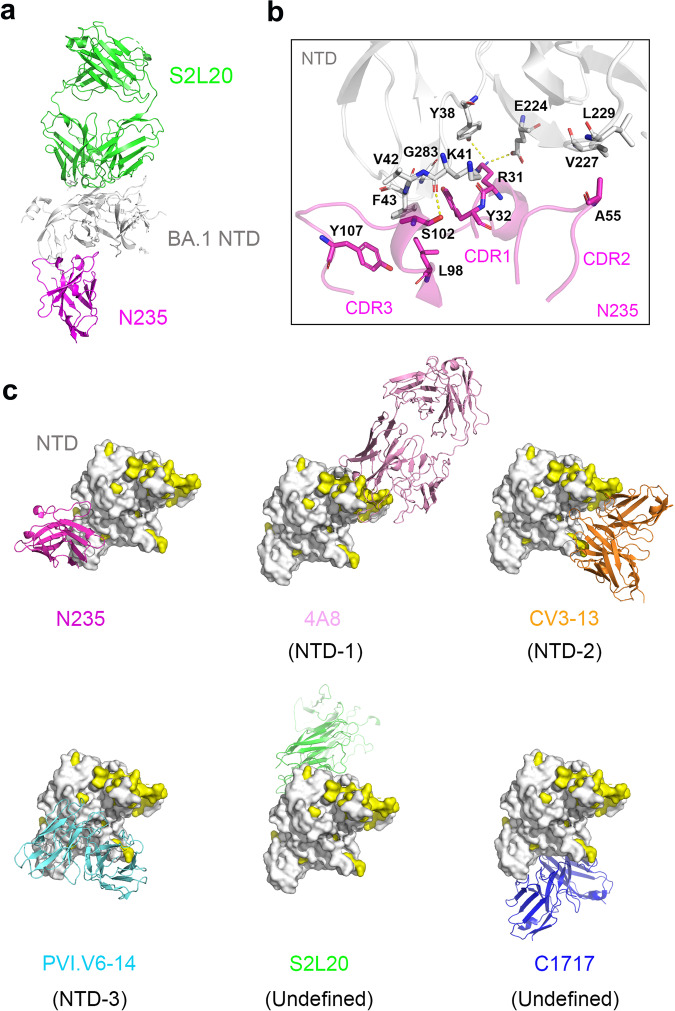
The complex structure of N235 bound to the NTD. **a** The overall structure of N235/S2L20/BA.1-NTD complex. **b** Structural details of N235 (magenta) binding to BA.1-NTD (gray). The structure is shown as a cartoon and residues involved in the hydrophobic interaction as sticks. H-bonds are presented as yellow dotted lines. **c** Comparison of epitope footprints between N235 (magenta) and previously reported antibodies on the NTD surface: 4A8 (pink, PDB: 8dm4), CV3-13 (orange, PDB: 7rq6), PVI.V6-14 (cyan, PDB: 7rbu), S2L20 (green) and C1717 (blue, PDB: 7uar). Mutations occurring in previously circulating VOCs (Alpha, Beta, Gamma and Delta) and currently circulating Omicron sub-variants (BA.1, BA.2, BA.2.75, BA.2.3.20, BA.3, BA.4, BA.5, BA.5.1.3, BQ.1, BQ.1.1, BF.7, XBB, XBB.1.5, EG.5, EG.5.1, BA.2.86 and JN.1) are presented in yellow

We then tried to resolve the epitope of nanobody N235 on the NTD surface. To make the mAb/NTD big enough for cryo-EM study, an NTD mAb, S2L20 was used to co-mix for N235/S2L20/BA.1-NTD complex (Supplementary Fig. 4), whose structure was determined at 2.8 Å resolution (Fig. 3a, Supplementary Table 1 and Supplementary Fig. 5). The relevant analyses of the interactions between nanobody N235 and NTD were conducted by CCP4 suit^25^ (Supplementary Table 2). It revealed that nanobody N235 bound to the NTD with its three CDRs (CDR1-3) and one framework region (FR, FR1). Of which, R31, Y32, A55, L98, S102 and Y107 within the three CDRs of nanobody N235 interacted with Y38, K41, V42, F43, E224, V227, L229 and G283 in the NTD (Fig. 3b). Specifically, R31 of nanobody N235 formed two hydrogen (H)-bonds with Y38 and E224 of the NTD, while S102 formed one H-bond with NTD-K41 (Fig. 3b). Notably, R31 also established a salt bridge with E224. Besides, structural superimposition of N235/S2L20/BA.1 NTD, 4A8/BA.2 NTD (PDB: 8dm4), CV3-13/PT NTD (PDB: 7rq6), PVI.V6-14/PT NTD (PDB: 7rbu) and C1717 (PDB: 7uar) showed that N235 recognized a cryptic epitope of NTD that previously uncharacterized (Fig. 3c), which merely overlapped with that of PVI.V6-14 at residues L226 and V227 (Supplementary Fig. 9c).

To validate the solved complex structure, besides the aforementioned BA.1 NTD Y38, K41 and E224 that formed strong hydrophilic interactions with N235, we also chose V42 and F43 due to their more than 10 Van der Walls contacts with N235 (Supplementary Fig. 6a). The SPR results demonstrated a reduced affinity in all tested NTD mutants, including BA.1 NTD-Y38A, -K41A, -V42A, -F43A, and -E224A with N235, confirming their contribution in the interaction with N235 (Supplementary Fig. 6b and h). Specifically, N235 exhibited a 10- to 100-fold reduction in affinity with mutants Y38A, K41A, V42A, and E224A, and there was a > 700-fold reduction in affinity between N235 and the F43A mutant, highlighting the crucial role of F43.

In addition, sequence analyses showed that N235 bound to the distinct epitope consisting of amino acid residues highly conserved across SARS-CoV-2 variants (Supplementary Fig. 7), which is consistent with its broad-spectrum binding capacity and neutralization activity (Fig. 1a and b). To assess the breadth of N235 against other coronaviruses (CoVs), we aligned the sequences of SARS-CoV-2 NTD with those of representative sarbecoviruses as well as other five human CoVs, namely MERS-CoV, HKU1, HCoV-OC43, HCoV-NL63, and HCoV-229E (Supplementary Fig. 8). The results revealed that SARS-CoV-2 NTD shares more than 44% sequence identity with eight sarbecoviruses in clade 1, more than 38% identity with five sarbecoviruses in clade 2, and more than 40% identity with two sarbecoviruses in clade 3. In terms of the N235 epitope, RaTG13 contains the same residues as SARS-CoV-2, suggesting its susceptibility to this nanobody. While, SARS-CoV NTD, which is another member in clade I containing 6 substitutions, was incapable of binding to N235 (Supplementary Fig. 3). In general, residues that are equivalent to N235 epitope in clade I exhibit the most conservation, followed by those in clade II and clade III CoVs. However, the sequence identity of SARS-CoV-2 NTD is lower than 48% when compared to the other five human CoVs, and most of the N235 epitope residues vary, which were consistent with their incapabilities to interact with N235 (Supplementary Fig. 8).

We then carried out the competitive binding assay (“checkerboard competition”) of nanobody N235 with some representative NTD-targeted antibodies (4A8, CV3-13, PVI.V6-14, S2L20 and C1717) previously reported. Consistent with their epitopes, N235 displayed competitive binding with PVI.V6-14 (Supplementary Fig. 9a and b), due to their overlapped binding at residues L226 and V227 (Supplementary Fig. 9c).

### N235 and MN235 induce S1 shedding from trimeric S protein

**Fig. 4:**
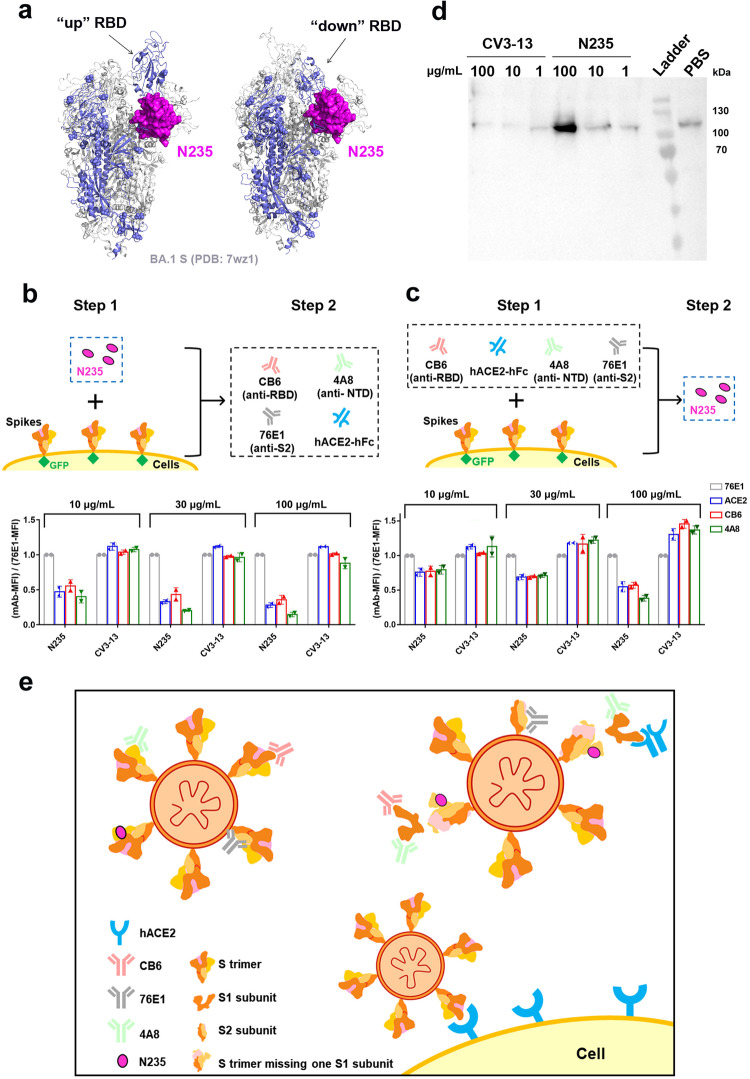
Interpretation of N235/MN235-mediated neutralization mechanism. **a** The overall features of N235 bound to the Omicron sub-variant BA.1 S trimer. The NTD/N235 complex was superimposed onto the S trimer (PDB code: 7wz1), which contains one “up” RBD and two “down” RBDs. The S monomer that clashed with N235 is colored in slate. **b** Comparison of mean fluorescence intensity (MFI). The GFP-fused S proteins were transiently expressed on the surface of BHK-21 cells and stained with His-tagged N235 or CV3-13 Fab in 10, 30, and 100 μg/mL, respectively. The complex proteins were then incubated with RBD-targeting antibody CB6, human ACE2 (hACE2), S2-targeting antibody 76E1, and NTD-targeting antibody 4A8. Experiments were performed twice, and one representative was displayed. **c** Comparisons of MFI. The GFP-fused S proteins were transiently expressed on the surface of BHK-21 cells and stained with RBD-targeting antibody CB6, S2-targeting antibody 76E1, and NTD-targeting antibody 4A8 before incubation with His-tagged N235 or CV3-13 Fab in 10, 30, and 100 μg/mL, respectively. The vertical axis represents the level of S1 shedding, calculated by dividing the MFI of cells with surface-expressed S treated with CB6, hACE2, 4A8 and 76E1 by the MFI of the 76E1-treated group. Cells were gated based on the FSC-A and SSC-A (P1) as shown in Supplementary Fig. 11c. **d** Western blot analysis of S1 subunits. The S proteins were transiently expressed on the surface of 293T cells and incubated with His-tagged N235 or CV3-13 Fab in 1, 10, and 100 μg/mL, respectively. PBS was negative control. The supernatants were collected after 1 h and detected via Western blot assay using an anti-SARS-CoV-2 S1 polyclonal antibody. Experiments were performed twice, and one representative was displayed. **e** Possible mechanism of N235-mediated neutralization: N235 binding triggers shedding off S1 subunits from S trimers, rending viruses non-infectious

When aligning the complex structure of N235/S2L20/NTD to the S protein (PDB: 7wz1), we found that the distinct epitopes recognized by N235 were located on the inner aspect of the NTD facing the adjacent RBD and were cryptic in the context of the entire trimeric S protein (Fig. 4a**)**. The epitope targeted by N235 in the S suggested an alternative mechanism for neutralization that is not solely reliant on direct inhibition of RBD-ACE2 binding. Based on blocking evaluation using flow cytometry experiments, it has been confirmed that N235 exerts neutralization independent of blocking S protein binding to ACE2 (Supplementary Fig. 10). Interestingly, N235 clashed with “up” or “down” RBD belonging to the adjacent protomer (Fig. 4a). We speculated that N235 might hinder the interaction between the NTD and the adjacent RBD, thereby favoring the dissociation of the trimeric S protein complex or inducing S1 subunit shedding from S proteins to achieve neutralization.

To test this hypothesis, we performed a binding assay using flow cytometry. The GFP-fused S proteins were transiently expressed on the surface of BHK-21 cells, stained with His-tagged N235 or CV3-13 Fab (as the evaluating antibodies), and then incubated with RBD-targeting antibody CB6,^4^ human Fc-tagged hACE2, S2-targeting antibody 76E1,^26^ and NTD-targeting antibody 4A8^15^ (as the indicated antibodies). When comparing the mean fluorescence intensity (MFI), we found that the binding of N235 influenced the NTD-directed antibody 4A8 and RBD-targeting antibody CB6 and hACE2 binding to S proteins. Similar results were also obtained when mAb S309, which interacts with both “up” and “down” RBDs, was used to stain the RBD (Supplementary Fig. 11). Furthermore, this effect became more pronounced with increasing concentrations of N235, indicating a concentration-dependent effect of N235 on blocking the binding of S1-targeting 4A8, CB6 and hACE2. While no decreased binding was observed between S2-targeting antibody 76E1 and S-expressing cells, which were pre-incubated with N235. As indicated in Fig. 3c, the binding epitope of N235 to the NTD was non-overlapped with that of 4A8. Neither N235 will block the interaction of RBD with CB6 or hACE2. One possible explanation should be the instability of the S trimer induced by the binding of N235. In other words, N235 appeared to trigger S1 subunit shedding or induce conformational changes via dissociating of S1 subunit more potently than control His-tagged CV3-13 Fab (Fig. 4b). Swapping the incubation order of the evaluating and the indicated antibodies obtained similar results: NTD-targeting antibody 4A8 bound to NTD, RBD-targeting antibody CB6 and S309, and hACE2 bound to RBD were removed after N235 binding (Fig. 4c and supplementary Fig. 11).

To further verify the shedding of S1 induced by N235, we then carried out a Western blot assay to test the S1 subunits in supernatants when S proteins that were expressed on the surface of 293T cells were incubated with His-tagged N235 or CV3-13 Fab in 1, 10, and 100 μg/mL, respectively. The results exhibited that the addition of N235 but CV3-13 at 100 μg/mL obviously increased the intensity of the band at around 110 kDa (Fig. 4d) that represented the S1 subunits.

**Fig. 5:**
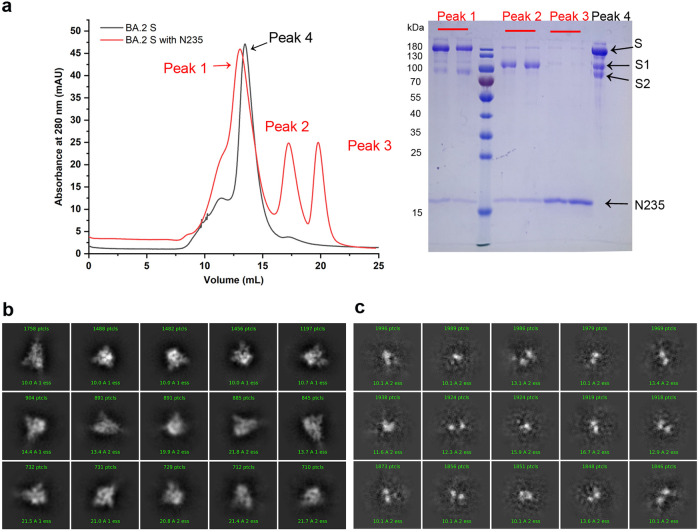
The effect of N235 on disrupting S trimer assembly. **a** Gel filtration profile of BA.2S proteins with and without N235. The BA.2S and N235 proteins elute as single monomer peaks in the gel filtration curves. The BA.2S/N235 complex displays a shifted complex peak. All the samples were assessed by SDS–PAGE. **b** 2D classifications of free BA.2S proteins, with the sample derived from the peak 4 as depicted in (**a**). **c** 2D classifications of complex protein of BA.2 S and N235, with the sample derived from the peak 1 as depicted in (**a**)

We then tried to test this mechanism using purified protein. We prepared the purified samples of BA.2S trimers incubated with or without the N235 and then subjected the samples to gel filtration for comparison. As displayed in Fig. 5a, in contrast with the free S protein with one sharp peak of the S trimer, three peaks were observed in the sample of BA.2S incubated with N235. The first one was the complex protein of BA.2S and N235, as indicated by the lower elution volume in the gel filtration and SDS-PAGE result. The second one was the shed S1 in the complex with N235, and the third one was the free N235 left. Additionally, we further observed the sample of peak 1 (BA.2S protein in complex with N235) by cryo-EM. Compared to those in the free S proteins (Fig. 5b), no intact S proteins were observed on the grids (Fig. 5c), indicating that N235 could disrupt S trimer assembly. Supportively, when the furin site was mutated in BA.1 or XBB (Δf), the S1-shedding effect caused by N235 was reduced (Supplementary Fig. 12).

We then performed a correlation analysis of the IC_50_s (μg/mL) of N235 with association rates (*k*_a_), dissociation rates (*k*_d_), and affinities (*K*_D_), respectively. The results indicated no significant correlations between IC_50_s to *k*_d_ and *K*_D_ (Supplementary Fig. 13). However, *k*_a_ seems to positively correlate with 1/IC_50_, suggesting stronger binding strength of N235 with NTD favors a higher prevention effect against pseudovirus transduction. Thus, the substantial results supported our proposed neutralization mechanism, in which N235 was more likely to neutralize the virus via antibody-induced S1 shedding (Fig. 4e), rendering S non-functional for viral entry.

Our next question is whether the MN235, which is the decametric form of N235, has a similar effect on the stability of the S trimer. Through flow cytometry-based experiments with dilution series of N235 and MN235, we found that IgM-like MN235 was able to induce S1 shedding from PT, BA.1 and XBB S proteins, which was similar to nanobody N235 under the same molar concentration of N235 moiety (Supplementary Fig. 14a), indicating MN235 could induce S1 shedding at the similar level to that of N235. Notably, a previous study suggested that IgM antibody prevents virus infection through the ligation of the viral particles,^27^ the transmission electron microscopy (TEM) results in this study also supported this effect (Supplementary Fig. 14b). The collective evidence from flow cytometry and TEM suggests that IgM-like MN235 mediates neutralization through mechanisms involving both cross-linking and inducing S1 shedding.

### MN235 displays effective prophylactic protection in vivo

Based on the effective neutralization in vitro, we evaluated the prophylactic protective efficacy of MN235 against Omicron sub-variant XBB in an adenovirus 5-expressing human ACE2 (Ad5-hACE2) mouse model. A single 5 or 20 mg/kg dose of MN235 was administered 2 h prior to XBB pseudovirus infection by the intranasal (i.n.) route to evaluate prophylactic efficacy. Mice receiving PBS were used as a control. Bioluminescence signal in the nasal passage was detected 24 h post-infection. We found that the bioluminescent signal was significantly reduced in mice that received 5 or 20 mg/kg MN235 by i.n. compared to mice that received PBS (Supplementary Fig. 15), indicating that MN235 can significantly prevent Omicron sub-variant XBB pseudovirus infection in vivo.

**Fig. 6:**
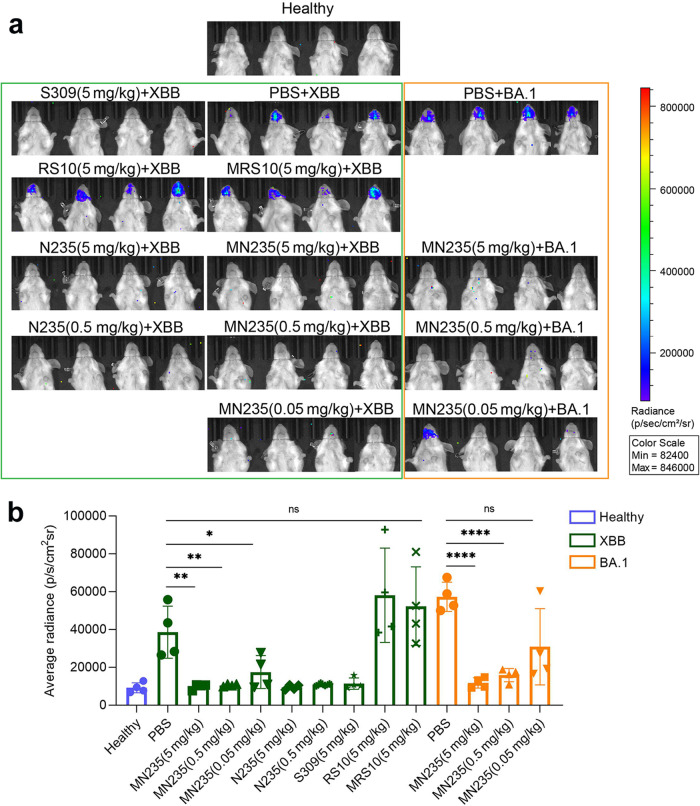
The prophylactic efficacy of MN235 and N235 against SARS-CoV-2 pseudovirus in vivo. **a** 6–8 week-old female BALB/c mice were infected by i.n. with Ad5‑hACE2. Five days later, mice were administered N235, MN235 and control antibody by i.n. before challenged with luciferase-expressing SARS-CoV-2 Omicron XBB and BA.1 pseudovirus (*n* = 4 per group). Mice were imaged 24 h after pseudovirus infection. **b** The bioluminescence signal in the nasal passage was quantified. Data are presented as mean ± s.d. Student’s *t*-test was used to analyze differences between groups. *P < 0.05

To explore the prophylactic effect of MN235 as well as N235 in-depth, we decreased the antibody doses in vivo. We also included mAb S309, which maintains neutralizing activity against XBB^8^ as the positive control, and an RSV F protein-targeting RS10 (developed in our lab) and its decameric MRS10 as negative controls. The results showed that S309 efficiently prevented the transduction of XBB pseudoviruses in vivo, while the RSV RS10 and anti-RSV IgM antibody MRS10 could not. Notably, both MN235 and N235 demonstrated effectivities against XBB pseudoviruses (Fig. 6). We also evaluated the efficacy of MN235 against BA.1 pseudovirus, and the results indicated its similar antiviral activity (Fig. 6). Our results highlight its potential as a promising antibody candidate for the prevention of COVID-19.

## Discussion

The widespread transmission of Omicron and its sub-variants across the globe, along with the reported resistance to antibody neutralization, poses a great challenge to the effectiveness of vaccines and therapeutic antibodies. Despite the multiple strategies considered to increase the efficacy of vaccines, the majority of mAbs aimed at blocking RBD binding to ACE2 have lost their effectiveness.^7,8,28^ Even Bebtelovimab (LY-CoV1404) has been revoked by the U.S. Food and Drug Administration (FDA) because it does not neutralize Omicron sub-variants BQ.1 and BQ.1.1 (https://www.fda.gov/). Antibodies directed to the RBD impose a selection pressure driving SARS-CoV-2 viral evolution, aiding immune evasion.^7,8^ Therefore, highly conserved epitopes in the non-RBD region are likely to be promising targets for vaccines and therapeutic antibodies.

Here, we discussed representative antibodies, including 4A8, CV3-13 and PVI.V6-14, from each of the three categorized NTD groups, as well as two antibodies, S2L20 and C1717, from the undefined groups. Recent studies have reported mutations in the NTD affecting the vulnerability of epitopes.^16,19,21,23^ For instance, the Y144 deletion in multiple Omicron sub-variants abrogated binding of 4A8 to NTD^21^; CV3-13 did not recognize the S protein from the Alpha VOC (also known as B.1.1.7)^19^; PVI.V6-14 almost completely lost its binding to the Gamma VOC (also known as P.1).^16^ These phenomena indicated that SARS-CoV-2 variants severely escaped the previously reported NTD-directed antibodies. In this study, we present an NTD-directed nanobody, N235, with broad cross-reactivity, and provide structural insights into a highly conserved cryptic epitope outside of the “supersite” of NTD across SARS-CoV-2 variants, including Delta, Omicron and its sub-variants.

However, the distinct epitope recognized by N235 is cryptic in the trimeric S protein, making it challenging to stimulate antibodies targeting this epitope during natural infection or when using trimeric S protein-based vaccines. This suggests that a separate NTD approach may be more efficient to stimulate broad-neutralizing antibodies.

Although the key NTD residues responsible for the interaction with N235 are highly conserved among SARS-CoV-2 variants evaluated in our study, the adjacent mutated residues might result in subtle changes in affinity. For instance, upon aligning the structures of F43 in the free PT NTD (PDB:7ddd) with that in the free BA.2.75 NTD (PDB: 8gs6), a significant 1.5 Å shift in the main chain was observed for BA.2.75 NTD compared to PT NTD (Supplementary Fig. 16). This observation may contribute to the different affinities and neutralization activities between different variants that N235 conferred, further suggesting a flexible or “plastic” structural characteristic of the NTD.

A previous study reported two SARS-CoV-2 RBD-directed antibodies 7D6 and 6D6 disrupting the viral S through structural clash with the adjacent NTD.^29^ Another RBD-directed antibody, P2C-1F11, has been reported to trigger shedding of the S1 subunit from the S to achieve neutralization.^30^ Through structural analyses, we speculated and then confirmed that N235 adopts a similar neutralization mechanism by inducing S1 subunit shedding from trimeric S. The positive correlation between *k*_a_ of N235 and its 1/IC_50_s (μg/mL) indicates that the higher binding strength to NTD facilitates stronger destruction of S proteins, further supporting the mechanism of N235.

Interestingly, with the similar transient expression level of S protein on the transfected cells as indicated by the GFP MFI, more S309-binding signals were observed in the PT sample than in BA.1 and XBB samples (supplementary Fig. 12a). Because S309 can interact with both “up” and “down” RBDs, and its binding strengths to PT, BA.1 and XBB are at a similar level.^8^ Thus, the different S309-binding MFIs probably indicate the different quantities of the RBD-containing S1 in the S protein, suggesting more intact S trimer on the PT samples than on the BA.1 and XBB samples. In other words, the BA.1 and XBB S trimers are probably more flexible, and both S1s are more easily shed, for example, after S binding to the receptors, which might contribute to the higher transmissibility of the Omicron sub-variants compared to the PT.

In our previous study, we reported the engineering of an IgM-like antibody, MR14, by fusing an RBD-directed nanobody, R14, with an IgM Fc domain. Despite showing similar binding affinities to the RBDs of the Omicron sub-variants BA.1, BA.2, BA.2.12.1, BA.2.75, and BA.3, as compared to R14, MR14 exhibited more than 2,000-fold enhancement in neutralization activity against these Omicron sub-variants.^6^ Based on that, the IgM-like MN235 was engineered via nanobody N235 fusing with conventional human IgM antibody Fc in this study. As expected, compared to monovalent N235, MN235 demonstrated a significant increase of more than 10-fold in neutralization with an IC_50_ range of 0.003–0.14 μg/mL against PT and the variants, including Delta, and Omicron sub-variants BA.1, BA.1.1, BA.2, BA.2.12.1, BA.2.75, BA.3, BA.4, BA.5, BQ.1.1 and BF.7. This neutralization capacity is similar to some cross-reactive RBD-directed antibodies, such as Beta-54.^8^ However, there was no significant improvement observed in neutralization against XBB, XBB.1.5, XBB.1.16, CH.1.1, EG.5, EG.5.1, BA.2.86 and JN.1. Our flow cytometry-based tests and TEM results indicated the IgM-like MN235 was able to induce S1 shedding at the similar level to that of N235 and to cause the cross-linking of the viral particles (Supplementary Fig. 14a). However, whether MN235 exerts different ligation effect on different variant particles needs further study. Additionally, whether other effects of the IgM antibody play roles in this different enhancement deserves further investigation.

Moreover, intranasal administration of MN235 with a low dose at 0.05 mg/kg can effectively prevent Omicron BA.1 and XBB pseudovirus infection in vivo. This construct could be a solution for the development of drugs via respiratory administration.

In conclusion, our study has confirmed the presence of a distinct and highly conserved cryptic epitope in the NTD, which is recognized by the nanobody N235. We have demonstrated that the IgM-like antibody MN235 exhibits effective antiviral activity both in vitro and in vivo. This research contributes to the emphasis on developing neutralizing antibodies beyond the RBD epitopes, enhancing our understanding of the antiviral mechanisms of NTD-targeting antibodies, and provides valuable insights into the “plastic” nature of the NTD. Importantly, our findings demonstrate the potential of these antibodies as promising drug candidates for respiratory administration against SARS-CoV-2 infection.

### Limitations of the study

In this study, N235 demonstrated the capability to disrupt trimer assembly, as evidenced by flow cytometry assays and Western blot analyses with S proteins expressed on cells. It is most likely that the features of S protein transiently expressed on the cells and those on the viruses/pseudoviruses envelope are the same, and N235 is supposed to exert a similar effect on the stability of S trimer on the virions or pseudovirus particles, which needs further studies.

## Materials and method

### Surface plasmon resonance (SPR) assays

The binding kinetics of N235 with NTDs from the SARS-CoV-2 PT and its variants (Alpha, Beta, Gamma, Delta), as well as Omicron sub-variants (BA.1, BA.2, BA.2.75, BA.2.3.20, BA.3, BA.5, BA.5.1.3, and XBB, et al.), were accessed using SPR analysis on a Biacore 8K system (GE Healthcare) with protein A chips (Cytiva Life Sciences) at 25 °C in single-cycle mode. All proteins used in the kinetic analysis were exchanged into the PBST buffer (2.7 mM KCl, 137 mM NaCl, 4.3 mM Na_2_HPO_4_, 1.4 mM KH_2_PO_4_, and 0.005% (v/v) Tween 20). Human Fc-tagged NTD proteins were captured using the protein A chip, and serial dilutions of His-tagged N235 were injected onto the chip surface to access binding. After each cycle, the sensor chips were regenerated using glycine (pH 1.7). The *K*_D_ values were calculated using Biacore 8K evaluation software (GE Healthcare). Figures were generated using OriginPro 9.1.

### Flow cytometry assay

The potential ability of the nanobody N235 to cause S1 shedding after binding to SARS-CoV-2 S was also assessed using flow cytometry. Initially, BHK-21 cells were transfected with pCAGGS vectors containing SARS-CoV-2 S fused to GFP and incubated for 48 h. Next, 2 × 10^5^ cells were collected, suspended in PBS, and incubated with 10, 30, or 100 µg/mL His-tagged nanobody N235 or control antibody Fab (CV3-13) at 37 °C for 60 min. Subsequently, cells were incubated with 10 µg/mL of hFc-tagged antibody or protein (ACE2, CB6, 4A8, 76E1) for 60 minutes at 37 °C. After two washes, cells were stained with anti-hFc/APC antibodies (Miltenyi) for 45 minutes at 37 °C. On the opposite, BHK-21 cells were transfected with pCAGGS vectors containing SARS-CoV-2 S fused with GFP and cultured for 48 h. Following this, 2 × 10^5^ cells were collected, suspended in PBS, and incubated with 10 µg/mL of hFc-tagged antibody or protein (ACE2, CB6, 4A8, 76E1) for 60 min at 37 °C. Then, cells were treated with 10, 30, or 100 µg/mL of His-tagged nanobody N235 or CV3-13 Fab (control) at 37 °C for 60 min. After two washes, the cells were stained with anti-hFc/APC antibodies (Miltenyi) for 45 min at 37 °C.

The potential of nanobody N235 to block the interaction between the SARS-CoV-2 S proteins and ACE2 was assessed by flow cytometry. BHK-21 cells were transfected with pEGFP-N1 vectors containing hACE2 and incubated for 48 h. Subsequently, His-tagged SARS-CoV-2 S protein (10 µg/mL) was incubated with human Fc-tagged nanobody N235 (2 µg/mL) at 4 °C for 30 min. The mixture was then added to BHK-21 cells (2 × 10^5^) and incubated at 37 °C for 1 h. After three washes with PBS, the cells were stained with anti-His/APC antibodies (BioLegend). In the negative control group, the cells were incubated with SARS-CoV-2 S and a previously reported antibody CV3-13,^19^ while in the positive control group, the cells were incubated with SARS-CoV-2 S with nanobody R14 ^6^ were used as positive control.

### Western blot analysis of S1 in the cell supernatant

The shedding of S1 protein into the supernatant was assessed by Western blot assay. Briefly, ~2 × 10^5^ 293T cells transfected with prototype S expression vector were treated with testing antibodies (N235 or CV3-13 at 1, 10, and 100 μg/mL) or PBS for 1 h at 37 °C. Subsequently, the S1 protein-containing supernatant was centrifuged to remove cell debris, separated on a 10% reducing SDS–PAGE gel, and then transferred onto polyvinylidene fluoride membranes. Immunoblotting was performed using an anti-SARS-CoV-2 S1 polyclonal antibody (1:2000 dilution; Sino Biological, Cat#40150-T62) followed by incubation with horseradish peroxidase (HRP)-conjugated goat anti-rabbit secondary antibody (1:10000 dilution; Gene-Protein Link, Cat# P12S12S).

### Engineering and generation of IgM versions of antibodies

The sequence encoding N235 fused to the human IgM Fc region was inserted into the pCAGGS vector to generate an IgM recombinant antibody (MN235). MN235 proteins were expressed by co-transfecting the recombinant MN235 plasmid with a vector expressing human J-chain into Freestyle 293F cells. The resulting MN235 proteins were purified by HiTrap™ IgM Purification HP (Cytiva) and Superose 6 Increase 10/300 GL (Cytiva Life Sciences) chromatography.

### Pseudovirus-based neutralization

To generate the SARS-CoV-2 pseudovirus, HEK293T cells expressing the S proteins of the SARS-CoV-2 prototype or its variants were infected with the rVSV-ΔG virus. After 30 h, the supernatants containing the pseudoviruses were harvested, centrifuged, and filtered through 0.45 μm membranes. For the neutralization assay, 1 × 10^4^ Vero cells were seeded in each well of a 96-well plate 24 h prior to infection. Nanobodies were serially diluted five-fold and incubated with an equal volume of pseudovirus supernatant containing 1000 fluorescence focus units (FFU) for 1 h at 37 °C. Nanobody dilutions were performed in triplicate. After 15 h, the number of infected cells was measured using a CQ1 Confocal Quantitative Image Cytometer (Yokogawa), and the half-maximal inhibitory concentration (IC_50_) was calculated using GraphPad Prism 7.0.

### Live virus neutralization

Neutralization assays with live virus were conducted based on cytopathic effect (CPE). Serial dilutions of MN235 (50 μL) were incubated with an equal volume of 100 50% tissue culture infectious dose (TCID_50_) of live viruses, including the PT, Delta, Omicron BA.1, BA.1.1, BA.2, BA.4, BA.5, BF.7, XBB, and EG.5.1 for 1 h at 37 °C. The mixtures were added to Vero cells in quadruplicate and incubated for 3 days at 37 °C. CPE was observed and recorded on day 4 after infection. IC_50_ values of the nanobodies were calculated using GraphPad Prism 7.0. All experiments were conducted within the BSL3 facility of the China CDC.

### Cryo-EM sample preparation and data acquisition for the N235/NTD complex

To explore the epitope of N235 in NTD, the nanobody N235, nanobody N36, S2L20 Fab, and NTD proteins of BA.1 were incubated together to prepare the complexes for building the cryo-EM structure model; however, the N36 component was not found in the complex by SDS-PAGE (Supplementary Fig. 4). The N235/S2L20-Fab/BA.1-NTD complex sample (4.0 μL, 0.4 mg/mL) was first applied onto a Cu Quantifoil 1.2/1.3 holey carbon grid that had been glow discharged for 40 s. Subsequently, the grid was then blotted with a blot time of 3 seconds and blot force 0 at a temperature of 4 °C and a humidity level of >98% before being plunge frozen in liquid ethane using a Vitrobot Mark IV (Thermo Fisher). The prepared grid was then transferred to a 300 kV Titan Krios TEM equipped with a Gatan K3 detector and GIF Quantum energy filter. Movies were collected at ×130,000 magnification with a calibrated pixel size of 0.54 Å over a defocus range of −1.0 to −2.0 μm in super-resolution counting mode, with a total dose of 60 e-/Å^2^ using EPU (Thermo Fisher Scientific) automated acquisition software.

### Image processing for the N235/NTD complex

For the N235/S2L20-Fab/BA.1-NTD complex, the detailed data-processing workflow is outlined in supplementary Fig. 5. All raw dose-fractionated image stacks underwent 2× binning, alignment, dose-weighting, and summation using MotionCor2.^31^ Subsequently, contrast transfer function (CTF) estimation, particle picking and extraction, 2D classification, ab initio model generation, and 3D refinements were conducted in cryoSPARC v.3.3.1. A dataset comprising 3240 micrographs of the complex was collected, yielding 1,251,507 initial particles that were selected and extracted with a box size of 512 pixels. After three rounds of iterative 2D classification, a clean set of 223,582 particles was utilized to generate four ab initio 3D reconstructions. Further refinement involved the application of heterogeneous refinement using the three dominant classes and particles associated with the four initial volumes. One volume, encompassing approximately ~68.4% of the total particles, was selected from the three 3D classes for non-uniform refinement, resulting in a final density map at 2.81 Å resolution, as estimated by the gold-standard Fourier shell correlation cut-off value of 0.143.

### Model building and structure refinement for the N235/NTD complex

For the initial model building of the N235/S2L20-Fab/BA.1-NTD complex, we employed the SARS-CoV-2 S trimer with S2L20 Fab (PDB code 7TM0) and nanobody 1D12 (PDB code 6V80) as the starting model, fitting them into the corresponding overall cryo-EM maps using UCSF Chimera v.1.15.^32^ Mutation and manual adjustment were carried out using Coot v.0.9.3,^33^ with most residue side chains being clearly visible on the map. Each residue underwent manual verification, considering its chemical properties during model building. Multiple rounds of real-space refinement in PHENIX-1.20.1^34^ and manual building in Coot were conducted until the final reliable models were achieved. Molprobity^35^ was utilized to validate the geometry and ensure the quality of the structure. Details regarding data collection, 3D reconstruction, and model building are provided in Table S1. Figures were prepared using Chimera and PyMol v.2.0.

### Cryo-EM sample preparation, data acquisition and image processing for the N235/BA.2S complex

The N235-BA.2-S complex sample (4.0 μL, 0.2 mg/ml) was first applied onto a Cu Quantifoil 1.2/1.3 holey carbon grid with 2 nm continuous carbon coating, which was then glow discharged for 25 s. Separately, the BA.2-S sample (4.0 μL, 1.2 mg/mL) was applied to a 1.2/1.3 Au C-Flat grid, previously glow discharged for 10 s at 15 mA. Subsequently, the grids were blotted at 4 °C and a humidity level of >98% before being plunge-frozen into liquid ethane using a Vitrobot Mark IV (Thermo Fisher).

The prepared grids were transferred to a 200 kV Titan Krios TEM equipped with a Falcon2 detector. Movies were captured at 120,000× magnification with a calibrated pixel size of 1.2 Å, spanning defocus range of −1.8 to −3.0 μm in super-resolution counting mode and employing a total dose of 50 e-/Å^2^ using EPU (Thermo Fisher Scientific) automated acquisition software.

Raw movies underwent motion correction using MotionCor2 v1.2.4,^31^ and micrograph contrast transfer function (CTF) correction parameters were estimated using CTFFIND 4.0 within cryoSPARC v.3.3.1. Reference-free particle picking was performed using Blob picker in cryoSPARC and then particles were picked and extracted for 2D classification. After two to three rounds of 2D classifications, we get the final results.

### Animal experiments with Omicron sub-variant BA.1 and XBB challenge

Animal studies involving the Omicron sub-variants BA.1 and XBB were conducted following approval from the Research Ethics Committee of the Institute of Microbiology, Chinese Academy of Sciences (APIMCAS2022124).

Female BALB/c mice aged 6–8 weeks were infected by i.n. with Ad5‑hACE2. After five days, mice were challenged with luciferase-expressing Omicron XBB or BA.1 pseudovirus (*n* = 4 per group) i.n. 2 h after administered intranasally with nanobody N235 and IgM-like MN235, alongside a positive control (mAb S309), a negative control nanobody targeting RSV (RS10), and a negative control IgM antibody specific to RSV (MRS10). Bioluminescence imaging was conducted 24 hours post-pseudovirus infection to quantify signal intensity in the nasal passage. Following D-luciferin administration, the mice were anaesthetized and imaged using IVIS® Lumina III (PerkinElmer) for pseudocolor visualization. Data are presented as mean ± standard deviation (s.d.), with statistical analysis performed using Student’s *t*-test (*P < 0.05).

### Supplementary information

Supplementary information in clear version

## Data Availability

Atomic coordinates and cryo-EM density maps corresponding to the NTD of SARS-CoV-2 BA.1 in complex with N235 (PDB ID: 8JVA; whole map: EMD-36672) have been submitted to the Protein Data Bank (www.rcsb.org) and the Electron Microscopy Data Bank (www.ebi.ac.uk/pdbe/emdb), respectively. Additionally, the data and materials employed in this current study are accessible upon reasonable request from the corresponding authors.

## References

[1] Yu D (2022). Coronavirus GenBrowser for monitoring the transmission and evolution of SARS-CoV-2. Brief. Bioinform..

[2] Wang Q (2020). Structural and functional basis of SARS-CoV-2 entry by using human ACE2. Cell.

[3] Lan J (2020). Structure of the SARS-CoV-2 spike receptor-binding domain bound to the ACE2 receptor. Nature.

[4] Shi R (2020). A human neutralizing antibody targets the receptor-binding site of SARS-CoV-2. Nature.

[5] Sulea T (2022). Structure-based dual affinity optimization of a SARS-CoV-1/2 cross-reactive single-domain antibody. PLoS ONE.

[6] Liu H (2023). Two pan-SARS-CoV-2 nanobodies and their multivalent derivatives effectively prevent Omicron infections in mice. Cell Rep. Med..

[7] Huang M (2022). Atlas of currently available human neutralizing antibodies against SARS-CoV-2 and escape by Omicron sub-variants BA.1/BA.1.1/BA.2/BA.3. Immunity.

[8] He Q (2023). An updated atlas of antibody evasion by SARS-CoV-2 Omicron sub-variants including BQ.1.1 and XBB. Cell Rep. Med..

[9] Ling Z, Yi C, Sun X, Yang Z, Sun B (2023). Broad strategies for neutralizing SARS-CoV-2 and other human coronaviruses with monoclonal antibodies. Sci. China Life Sci..

[10] Wang Q (2023). Antigenicity and receptor affinity of SARS-CoV-2 BA.2.86 spike. Nature.

[11] He, Q. et al. Neutralization of EG.5, EG.5.1, BA.2.86, and JN.1 by antisera from dimeric receptor-binding domain subunit vaccines and 41 human monoclonal antibodies. *Med* (2024). 10.1016/j.medj.2024.03.006 38574739

[12] Li Y (2021). Exploring the regulatory function of the N-terminal domain of SARS-CoV-2 spike protein through molecular dynamics simulation. Adv. Theory Simul..

[13] Zhou H (2019). Structural definition of a neutralization epitope on the N-terminal domain of MERS-CoV spike glycoprotein. Nat. Commun..

[14] Guo H (2022). The glycan-binding trait of the sarbecovirus spike N-terminal domain reveals an evolutionary footprint. J. Virol..

[15] Chi X (2020). A neutralizing human antibody binds to the N-terminal domain of the Spike protein of SARS-CoV-2. Science.

[16] Altomare CG (2022). Structure of a vaccine-induced, germline-encoded human antibody defines a neutralizing epitope on the SARS-CoV-2 spike N-terminal domain. mBio.

[17] McCallum M (2022). Structural basis of SARS-CoV-2 Omicron immune evasion and receptor engagement. Science.

[18] Hastie KM (2021). Defining variant-resistant epitopes targeted by SARS-CoV-2 antibodies: a global consortium study. Science.

[19] Beaudoin-Bussieres G (2022). A Fc-enhanced NTD-binding non-neutralizing antibody delays virus spread and synergizes with a nAb to protect mice from lethal SARS-CoV-2 infection. Cell Rep..

[20] Rosa A (2021). SARS-CoV-2 can recruit a heme metabolite to evade antibody immunity. Sci. Adv..

[21] McCallum M (2021). N-terminal domain antigenic mapping reveals a site of vulnerability for SARS-CoV-2. Cell.

[22] Wang Z (2022). Analysis of memory B cells identifies conserved neutralizing epitopes on the N-terminal domain of variant SARS-Cov-2 spike proteins. Immunity.

[23] Cao Y (2022). Characterization of the enhanced infectivity and antibody evasion of Omicron BA.2.75. Cell Host Microbe.

[24] Chi X (2023). Comprehensive structural analysis reveals broad-spectrum neutralizing antibodies against SARS-CoV-2 Omicron variants. Cell Discov..

[25] Potterton E, Briggs P, Turkenburg M, Dodson E (2003). A graphical user interface to the CCP4 program suite. Acta Crystallogr. D.

[26] Sun X (2022). Neutralization mechanism of a human antibody with pan-coronavirus reactivity including SARS-CoV-2. Nat. Microbiol..

[27] Singh T (2022). A Zika virus-specific IgM elicited in pregnancy exhibits ultrapotent neutralization. Cell.

[28] Cao Y (2022). BA.2.12.1, BA.4 and BA.5 escape antibodies elicited by Omicron infection. Nature.

[29] Li T (2021). Cross-neutralizing antibodies bind a SARS-CoV-2 cryptic site and resist circulating variants. Nat. Commun..

[30] Ge J (2021). Antibody neutralization of SARS-CoV-2 through ACE2 receptor mimicry. Nat. Commun..

[31] Zheng SQ (2017). MotionCor2: anisotropic correction of beam-induced motion for improved cryo-electron microscopy. Nat. Methods.

[32] Pettersen EF (2004). UCSF Chimera-a visualization system for exploratory research and analysis. J. Comput. Chem..

[33] Emsley P, Cowtan K (2004). Coot: model-building tools for molecular graphics. Acta Crystallogr. D.

[34] Adams PD (2010). PHENIX: a comprehensive Python-based system for macromolecular structure solution. Acta Crystallogr. D Biol. Crystallogr..

[35] Williams CJ (2018). MolProbity: more and better reference data for improved all-atom structure validation. Protein Sci..

