# Supplementary material for: Enhanced potency of an IgM-like nanobody targeting conserved epitope in SARS-CoV-2 spike N-terminal domain
Source: Signal Transduct Target Ther. 2024 May 13;9:131. doi: 10.1038/s41392-024-01847-8 (PMC11091055; doi:10.1038/s41392-024-01847-8)
Supplement: Supplementary file 1 — Supplementary information in clear version [file 41392_2024_1847_MOESM1_ESM.docx]

Supplementary Materials for

Enhanced potency of an IgM-like nanobody targeting conserved epitope in SARS-CoV-2 spike N-terminal domain

Bo Liu^1,2,#^, Honghui Liu^2,#^, Pu Han^2,#^, Xiaoyun Wang^2,#^, Chunmei Wang^2,3^, Xinxin Yan^1,2^, Wenwen Lei^4^, Ke Xu^4^, Jianjie Zhou^2^, Jianxun Qi^2,5^, Ruiwen Fan^1^, Guizhen Wu^4,*^, Wen-xia Tian^1,*^, George F. Gao^1,2,*^, Qihui Wang^1,2,6,*^

Correspondence to: [wugz@ivdc.chinacdc.cn](mailto:wugz@ivdc.chinacdc.cn) (G. W.),[wenxiatian@126.com](mailto:wenxiatian@126.com) (W. T.), [gaof@im.ac.cn](mailto:gaof@im.ac.cn) (G. F. G.), [wangqihui@im.ac.cn](mailto:wangqihui@im.ac.cn) (Q. W.).

**This PDF file includes:**

Materials and Methods

Supplementary Text

Figures. S1 to S16

Tables S1 to S2

Materials and Methods

Cell and viral sources

HEK293T (ATCC CRL-3216), Vero (ATCC CCL81), and BHK-21 cells (ATCC CCL-10) were cultured in Dulbecco’s Modified Eagle’s medium (DMEM) supplemented with 10% fetal bovine serum at 37 °C in a humidified incubator with 5% CO_2_. Freestyle 293F cells were cultured in SMM 293-TII medium at 37 °C in a shaking incubator with 5% CO_2_. The SARS-CoV-2 strains BetaCoV/Wuhan/IVDC-HB-envF13/2020 (GISAID: EPI_ISL_408511), Delta (NPRC 2.192100004), BA.1 (NPRC 2.192100007), BA.1.1 (NPRC 2.192100005), BA.2 (NPRC 2.192100010), BA.4 (NPRC 2.192100012), BA.5 (NPRC 2.192100014), BF.7 (NPRC 2.192100019), XBB (NPRC 2.192100020) and EG.5.1 (NPRC 2.192100031) were isolated by the National Institute for Virus Disease Control and Prevention (Chinese Center for Disease Control and Prevention, China CDC).

Protein expression

The coding sequences of C-terminal His-tagged NTD and S from the SARS-CoV-2 prototype and Omicron sub-variant BA.1 were cloned into pCAGGS vectors. The coding sequences of nanobodies with a His-tag were also cloned into pCAGGS vectors. Recombinant plasmids were transfected into Freestyle 293F cells for protein expression. After five days, the supernatant containing soluble protein was harvested and subjected to purification using a HisTrap EXCEL 5-mL column (Cytiva Life Sciences). The protein underwent additional purification via gel filtration chromatography using a Superdex 200 Increase 10/300 GL column (Cytiva Life Sciences) or Superose 6 Increase 10/300 GL column (Cytiva Life Sciences). This process was carried out in a buffer solution containing 150 mM NaCl and 20 mM Tris-HCl (pH 8.0).

In SPR assays, Freestyle 293F cells were employed for the expression of human IgG Fc-tagged NTDs from SARS-CoV-2 prototype (PT) and its variants, including Alpha, Beta, Gamma, Delta, Mu, Lambda, as well as Omicron sub-variants (BA.1, BA.2, BA.2.75, BA.2.3.20, BA.3, BA.5, BA.5.1.3, XBB, XBB.1.5, XBB.1.16, BA.2.86, and EG.5.1)

Nanobody generation

The SARS-CoV-2 prototyped NTD and S proteins with a C-terminal His-tag were used for immunization in alpaca. The immunization process followed approved guidelines from the Animal Ethics Committee [2017(55)] at Shanxi Agricultural University (Taigu, Shanxi, China). Two female alpacas, aged 3 years, were immunized subcutaneously. One alpaca received 200 µg of SARS-CoV-2 NTD on days 0, 14, 28, and 42, while the other received 200 µg of SARS-CoV-2 S on days 0, 7, 28, 42, and 56. Blood samples were collected one week after the final immunization. *E. coli* (TG1), phage (VSCM13), and plasmid (pMES4) were kindly provided by Dr. Huilin Wang. The peripheral blood mononuclear cells (PBMCs) were isolated following the manufacturer’s instructions (Solarbio, Cat# P8610). Subsequently, the isolated PBMCs were suspended in 1 mL of TRIzol for total RNA extraction, following the protocol outlined by Invitrogen. Using the extracted total RNA as a template, the Invitrogen SuperScript Ⅱ First-Strand Synthesis System for RT-PCR was used to amplify the cDNA with random primers. Then, using cDNA as a template, the sequences of nanobodies were amplified with specific primers using nested PCR and cloned into the pMES4 vector. Recombinant pMES4 vectors were transformed into *E. coli* (TG1) cells using an electroporator to construct the library. SARS-CoV-2 prototyped NTD and S proteins were used as antigens for the biopan-specific phages. Randomly selected single colonies from each round of panning were tested using phage ELISA. Sequencing was conducted on positive clones to determine the nanobodies’ sequences. The sequences of the nanobodies with a C-terminal His-tag were subsequently cloned into the pCAGGS vectors. The recombinant nanobodies were then expressed in Freestyle 293F cells and purified following the aforementioned procedure.

Flow cytometry assay

Flow cytometry was employed to assess the interaction between the nanobodies and SARS-CoV-2. Briefly, BHK-21 cells were transfected with pCAGGS vectors containing SARS-CoV-2 S fused with GFP. Subsequently, after 48 hours, 2×10^5^ cells were collected, suspended in PBS, and exposed to the supernatants containing His-tagged nanobodies at 37 °C for 45 minutes. Following incubation, cells were washed twice and were then stained with anti-His/APC antibodies (Miltenyi) for 45 minutes at 37 °C. For experimental controls, cells incubated with antibodies (4A8, CV3-13, PVI.V6-14) targeting to SARS-CoV-2 were used as positive controls (PC), cells incubated with an irrelevant nanobody R23 acted as negative controls (NC), while cells treated only with PBS served as blank controls (BC).

For further evidence of N235’s mechanism in inducing S1 shedding, the GFP-fused S proteins of PT, BA.1 and XBB, as well as ones with inactive furin site (Δf) were transiently expressed on BHK-21 cell surfaces and then incubated with His-tagged N235, respectively. The complex proteins were then incubated with RBD-targeting antibody S309, and labeled with anti-hFc/APC antibodies. Experiments were performed twice.

For the neutralization mechanism of MN235 via inducing S1 shedding, the GFP-fused S proteins of PT, BA.1 and XBB were transiently expressed on BHK-21 cell surfaces and incubated with His-tagged N235 or MN235 in dilution series, respectively. Then the complexes were incubated with RBD-targeting antibody S309 and stained with anti-hFc/APC antibodies.

The potential of nanobody N235 to block the interaction between the SARS-CoV-2 S proteins and ACE2 was assessed by flow cytometry. BHK-21 cells were transfected with pEGFP-N1 vectors containing hACE2 and incubated for 48 hours. Subsequently, His-tagged SARS-CoV-2 S protein (10 µg/mL) was incubated with human Fc-tagged nanobody N235 (2 µg/mL) at 4 ℃ for 30 minutes. The mixture was then added to BHK-21 cells (2×10^5^) and incubated at 37 ℃ for 1 h. After three washes with PBS, the cells were stained with anti-His/APC antibodies (BioLegend). In negative control group, the cells were incubated with SARS-CoV-2 S and a previously reported antibody CV3-13, while in positive control group, the cells were incubated with SARS-CoV-2 S with nanobody R14 were used as positive control.

Surface plasmon resonance (SPR) assays

The binding affinities of N235 with human Fc-tagged BA.1 NTD and its mutants (BA.1 NTD-Y38A, -K41A, -V42A, -F43A, and -E224A) were determined using SPR analysis. Briefly, supernatants of human Fc-tagged NTD proteins were captured on the protein A chip, and serial dilutions of His-tagged N235 were injected onto the chip surface for test binding. After each cycle, the sensor chips were regenerated using glycine (pH 1.7). The *K*_D_ values were calculated using Biacore 8K evaluation software (GE Healthcare). Figures were generated using OriginPro 9.1.

The competition assays were evaluated using SPR analysis using a Biacore 8K system (GE Healthcare) with SA chips (Cytiva Life Sciences) at 25 °C in tandem using dual mode. Biotinylated SARS-CoV-2 NTD was captured at 10 μg/mL. The first antibody was captured for 420 s, and the second antibody (containing the first antibody) was captured for 150 s. The numbers refer to the shift values for each pair of interactions were calculated using Biacore 8K evaluation system (GE Healthcare), and figures were generatedusing GraphPad Prism 7.0.

Pseudovirus-based neutralization

To generate the SARS-CoV-2 pseudovirus, HEK293T cells expressing the S proteins of the SARS-CoV-2 prototype or its variants were infected with the rVSV-ΔG virus. After 30 hours, the supernatants containing the pseudoviruses were harvested, centrifuged, and filtered through 0.45 μm membranes. For the neutralization assay, 1×10^4^ Vero cells were seeded in each well of a 96-well plate 24 hours prior to infection. Nanobodies were serially diluted five-fold and incubated with an equal volume of pseudovirus supernatant containing 1,000 fluorescence focus units (FFU) for 1 hour at 37 °C. Nanobody dilutions were performed in triplicate. After 15 hours, the number of infected cells was measured using a CQ1 Confocal Quantitative Image Cytometer (Yokogawa), and the half-maximal inhibitory concentration (IC_50_) was calculated using GraphPad Prism 7.0.

Negative-stain assay by transmission electron microscopy (TEM)

Negative staining of the S protein and nanobody was conducted using TEM. The S protein and nanobody were incubated on ice for 24 hours, and then appropriately diluted with PBS. Subsequently, 10 μL of the sample was applied to a glow-discharged grid. After 1 minute, excess sample was removed by blotting the edge of the grid with filter paper. The grid was then quickly touched to a drop of ddH2O, and excess water was removed by blotting with filter paper. Finally, the grid was stained with 1% uranyl acetate, and TEM imaging was performed using a JEM-1400 instrument (JEDL, Japan) operated at an acceleration voltage of 80 kV.

Cryo-EM sample preparation and data acquisition for the N235/NTD complex

To explore the epitope of N235 in NTD, the nanobody N235, nanobody N36, S2L20 Fab, and NTD proteins of BA.1 were incubated together to prepare the complexes for building the cryo-EM structure model; however, the N36 component was not found in the complex by SDS-PAGE **(supplementary Fig4)**. The N235/S2L20-Fab/BA.1-NTD complex sample (4.0 μL, 0.4 mg/mL) was first applied onto a Cu Quantifoil 1.2/1.3 holey carbon grid that had been glow discharged for 40 s. Subsequently, the grid was then blotted with a blot time of 3 seconds and blot force 0 at a temperature of 4 °C and a humidity level of >98%, before being plunge frozen in liquid ethane using a Vitrobot Mark IV (Thermo Fisher). The prepared grid was then transferred to a 300 kV Titan Krios TEM equipped with a Gatan K3 detector and GIF Quantum energy filter. Movies were collected at 130,000× magnification with a calibrated pixel size of 0.54 Å over a defocus range of -1.0 μm to -2.0 μm in super-resolution counting mode, with a total dose of 60 e-/Å2 using EPU (ThermoFisher Scientific) automated acquisition software.

Image processing for the N235/NTD complex

For the N235/S2L20-Fab/BA.1-NTD complex, the detailed data-processing workflow is outlined in **supplementary Fig5**. All raw dose-fractionated image stacks underwent 2× binning, alignment, dose-weighting, and summation using MotionCor2. Subsequently, contrast transfer function (CTF) estimation, particle picking and extraction, 2D classification, ab initio model generation, and 3D refinements were conducted in cryoSPARC v.3.3.1. A dataset comprising 3,240 micrographs of the complex was collected, yielding 1,251,507 initial particles that were selected and extracted with a box size of 512 pixels. After three rounds of iterative 2D classification, a clean set of 223,582 particles was utilized to generate four ab initio 3D reconstructions. Further refinement involved the application of heterogeneous refinement using the three dominant classes and particles associated with the four initial volumes. One volume, encompassing approximately ~68.4% of the total particles, was selected from the three 3D classes for non-uniform refinement, resulting in a final density map at 2.81 Å resolution, as estimated by the gold-standard Fourier shell correlation cut-off value of 0.143.

Model building and structure refinement for the N235/NTD complex

For the initial model building of the N235/S2L20-Fab/BA.1-NTD complex, we employed the SARS-CoV-2 S trimer with S2L20 Fab (PDB code 7TM0) and nanobody 1D12 (PDB code 6V80) as the starting model, fitting them into the corresponding overall cryo-EM maps using UCSF Chimera v.1.15. Mutation and manual adjustment were carried out using Coot v.0.9.3, with most residue side chains being clearly visible on the map. Each residue underwent manual verification considering its chemical properties during model building. Multiple rounds of real-space refinement in PHENIX-1.20.1 and manual building in Coot were conducted until the final reliable models were achieved. Molprobity was utilized to validate the geometry and ensure the quality of the structure. Details regarding data collection, 3D reconstruction, and model building are provided in Table S1. Figures were prepared using Chimera3 and PyMol v.2.0.

Animal experiments with Omicron sub-variant BA.1 and XBB challenge

Animal studies involving the Omicron sub-variants BA.1 and XBB were conducted following approval from the Research Ethics Committee of Institute of Microbiology, Chinese Academy of Sciences (APIMCAS2022124).

Female SPF BALB/c mice aged 6-8 weeks were purchased from Vital River (Beijing, China). To establish infection susceptibility, the mice received intranasal (i.n.) administration of 8 × 10^9^ vp recombinant adenovirus 5 expressing human ACE2 (Ad5-hACE2) in 50 μL sterile PBS. After a five-days interval, mice were given MN235 (5 or 20 mg/kg body weight) or PBS (negative control) i.n. 2 h before being treated with 7.5 × 10^4^ TCID_50_ of XBB pseudovirus i.n. Healthy untreated mice served as controls. Bioluminescence was assessed 24 hours post-infection. Following D-luciferin administration, the mice were anesthetized and imaged using IVIS® Lumina III (PerkinElmer) for pseudocolor visualization. Data are presented as mean ± standard deviation (s.d.), with statistical analysis performed using Student’s t-test (**P* < 0.05).

Supplementary Text

Data availability

Atomic coordinates and cryo-EM density maps corresponding to the NTD of SARS-CoV-2 BA.1 in complex with N235 (PDB ID: 8JVA; whole map: EMD-36672) have been submitted to the Protein Data Bank (www.rcsb.org) and the Electron Microscopy Data Bank (www.ebi.ac.uk/pdbe/emdb), respectively. Additionally, the data and materials employed in this current study are accessible upon reasonable request from the corresponding authors.

Acknowledgements

We thank Zheng Fan (Institutional Center for Shared Technologies and Facilities, Institute of Microbiology of the Chinese Academy of Sciences, IMCAS) and Yuanyuan Chen (Institute of Biophysics, CAS) for technical help with Biacore experiments. We thank Jingnan Liang (Institutional Center for Shared Technologies and Facilities, IMCAS) for technical help with negative-stain experiments by transmission electron microscopy (TEM). We thank Xiaomei Li, Yuqian Mi, Xingxing Bai, and Erqi Pang at the Cryo-EM Center, Shanxi Academy of Advanced Research and Innovation for their technical support on the cryo-EM. We thank Xiaoyu Rong (Wenzhou Medical University) for supporting pseudovirus-based neutralization assay and flow cytometry assay. We thank Qingwen He (Zhejiang University), Shufan Yu (IMCAS) and Bin Bai (IMCAS) for supporting on protein purifications. We thank Dr. Zhennan Zhao (IMCAS) for supporting on cryo-EM. We thank Kun Xu (Beijing Institutes of Life Science, CAS) for generous support of Ad5-hACE2. We also thank professor Weijin Huang (National Institute for Food and Drug Control) for providing luciferase-expressed pseudoviruses. This work was supported by the National Key R&D Program of China (2022YFC2303403) and National Science Fund for Distinguished Young Scholars (82225021). Q.W. was also supported by the Young Scientists in Basic Research (YSBR-010).

Declaration of interests

Q. W., G. F. G., H. L., B. L. and J. Q. are listed as inventors on patent applications for N235 and MN235 based antiviral treatment. The other authors declare that they have no competing interests.

Author Contributions

Q. W. and G. F. G. designed this study. R. F. conducted alpaca immunization. H. L. selected the nanobody N235 by phage display technology. B. L., C. W., X. W., and J. Z. performed protein expression. B. L. and X. W. performed flow cytometry assays. B. L. and X. Y. tested the affinity of N235 to SARS-CoV-2 and its variants by SPR. H. L. and C. W. tested the affinity of N235 to BA.1 NTD with point mutations. B. L. and C. W. performed neutralization assay of pseudotyped virus. With the help of G. W., K. X. and W. L. performed neutralization assay of live virus in BSL-3 facility. B. L. and X. W. prepared the complex sample for cryo-EM. J. Q. and P. H. built structural model. H. L. and B. L. analyzed the complex structure. B. L., H. L., X. W. and X. Y. performed the animal experiment. B. L., H. L., X. W., C. W., and Q. W. analyzed the data. H. L., B. L., P. H., and J. Z. wrote the manuscript. Q. W., G. F. G., G. W., and W. T. revised the manuscript with other co-authors.


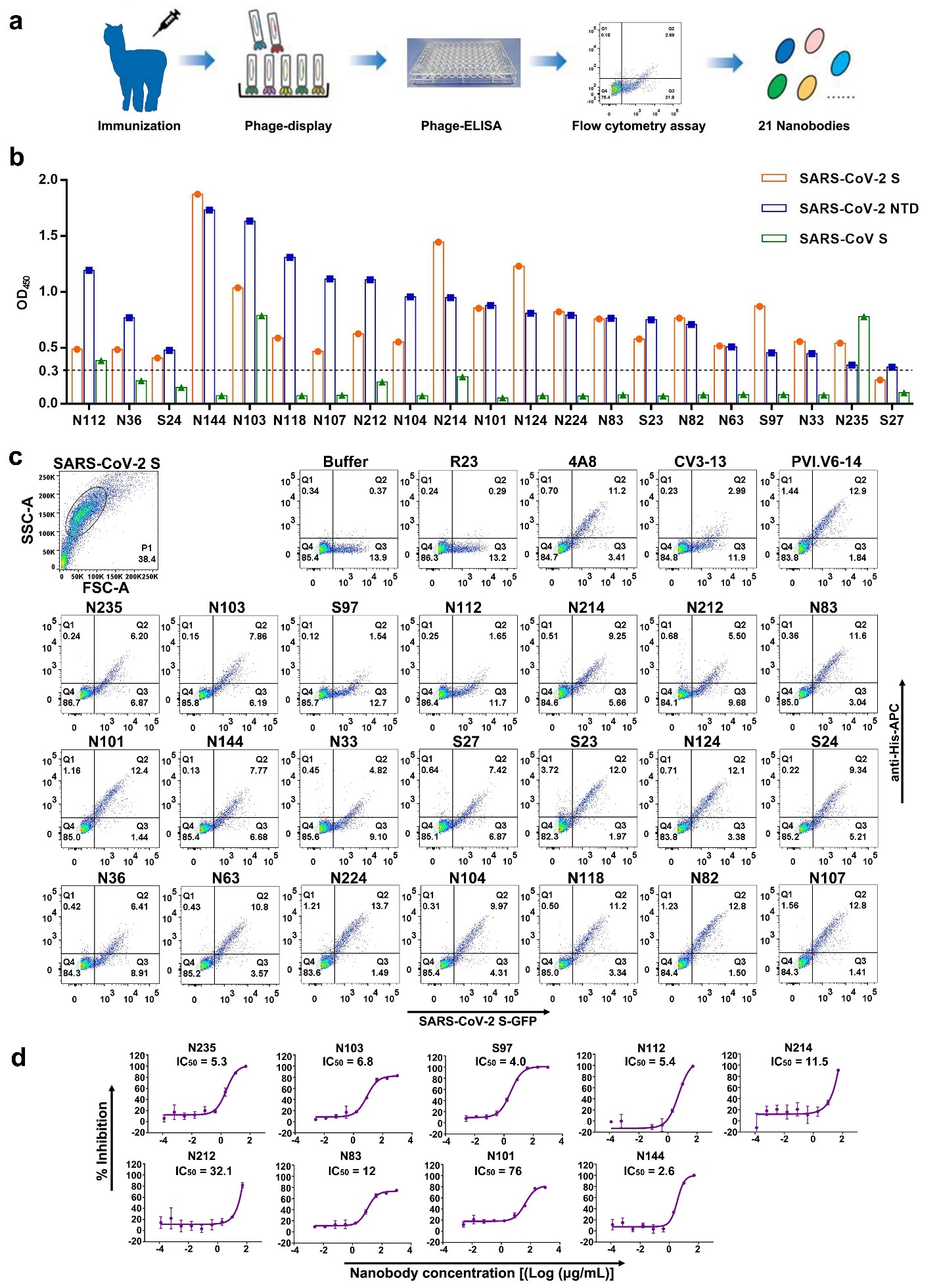


Figure. S1.

The generation and determination of nanobodies. (a) Overview of the nanobody generation process. (b) The determination of phage-ELISA coated with SARS-CoV-2 prototyped S proteins, NTD proteins and SARS-CoV S proteins. Above the dotted line are positive clones. (c) Binding determination between nanobodies and SARS-CoV-2 by a FACS-based assay. GFP-fused SARS-CoV-2 S protein was transiently expressed on the surface of BHK-21 cells, and strained with His-tagged antibodies. ‘NC’ presents negative control that was performed without nanobody protein. Positive control that was performed with previously reported antibodies that can bind to SARS-CoV-2 S protein, including irrelevant nanobody R23, His-tagged NTD-directed Fabs from 4A8, CV3-13 and PVI.V6-14. Then flow cytometry was conducted using BD FACSCanto. Cells were gated based on the FSC-A and SSC-A (P1). ‘SARS-CoV-2 S’ represents that PBS was incubated with BHK-21 cells expressing SARS-CoV-2 S protein and stained with anti-His-APC. (d) Neutralization curves of nanobodies against pseudotyped SARS-CoV-2 prototype in Vero cells in vitro. Experiments were independently repeated twice with similar results, and one representative curve is displayed. The IC_50_ values (μg/mL) from duplicates are presented as average.


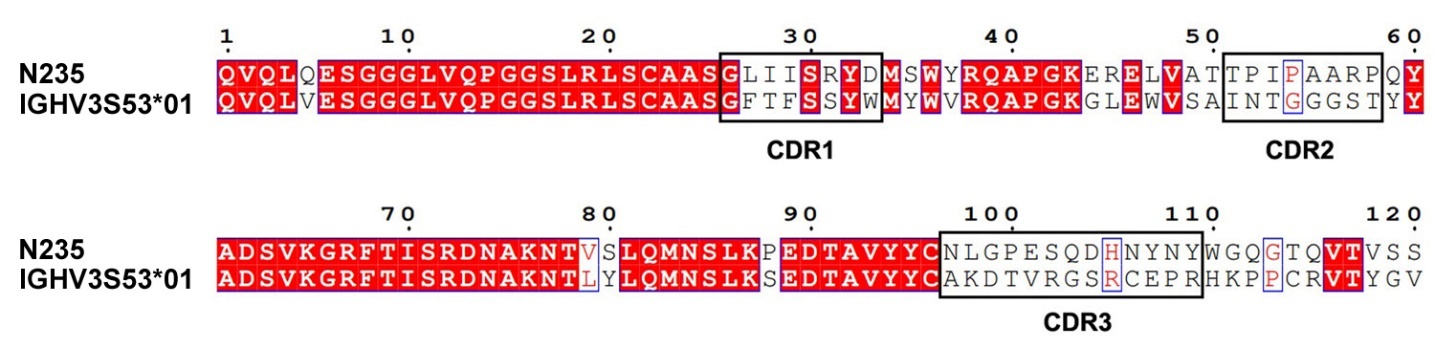


Figure. S2.

Sequence alignment analysis of nanobody N235 with the most similar alpaca V gene. Sequence alignment of nanobody N235 and the most similar alpaca V genes for heavy chain of antibodies from IMGT database (https://www.imgt.org/). The CDR region of the antibody is shown in the black box.


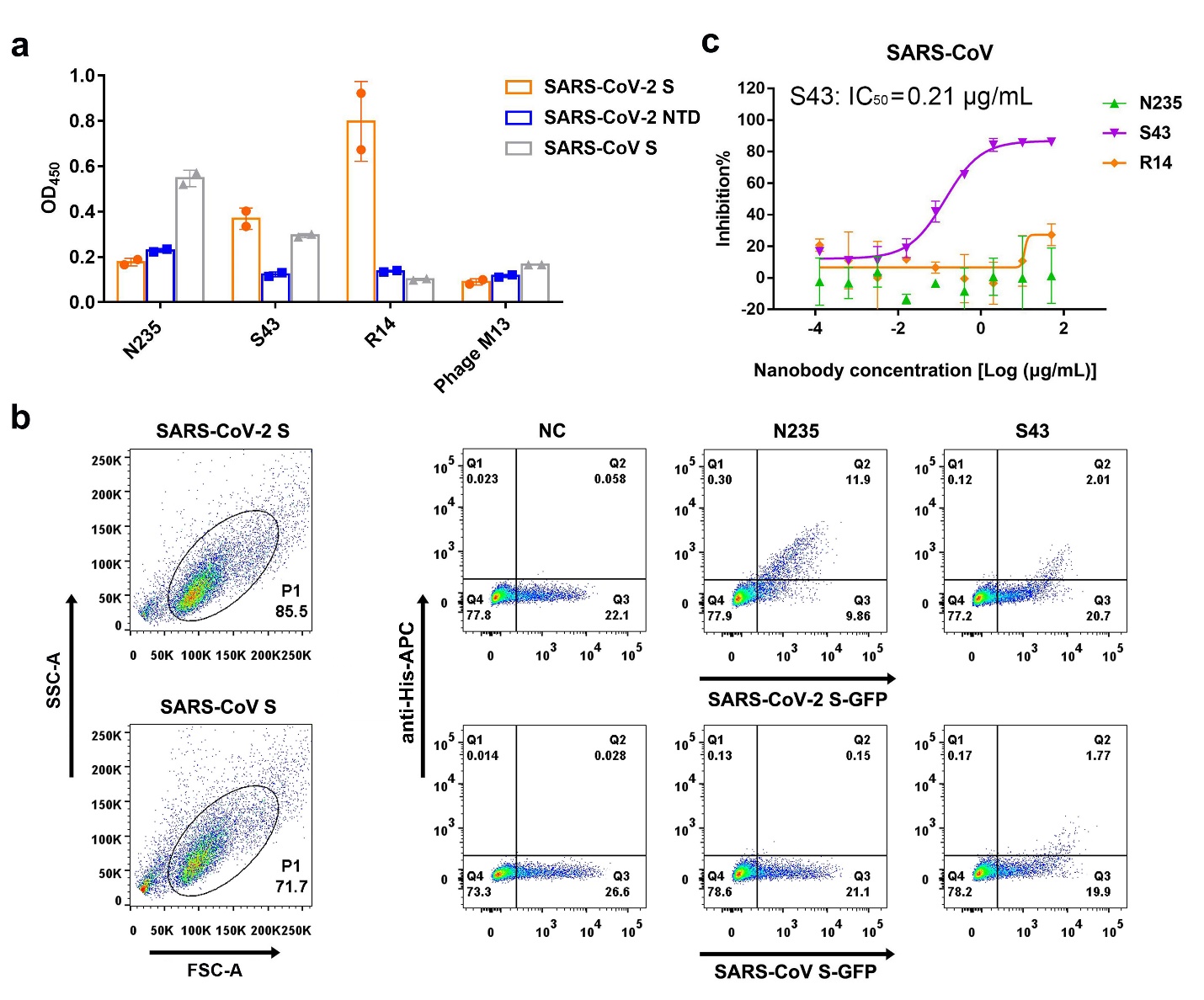


Figure. S3.

The assays of nanobody N235 binding to coronaviruses (CoVs). (a) The repeated determination of phage-ELISA coated with SARS-CoV-2 prototyped S proteins, NTD proteins and SARS-CoV S proteins. (b) Binding determination between nanobody N235 and SARS-CoV by a FACS-based assay. GFP-fused SARS-CoV-2 and SARS-CoV S protein was transiently expressed on the surface of BHK-21 cells, and strained with His-tagged antibodies. ‘NC’ presents negative control that was performed without nanobody protein. Then flow cytometry was conducted using BD FACSCanto. Cells were gated based on the FSC-A and SSC-A (P1). ‘SARS-CoV-2 S’ represents that PBS was incubated with BHK-21 cells expressing SARS-CoV-2 S protein and stained with anti-His-APC. ‘SARS-CoV S’ represents that PBS was incubated with BHK-21 cells expressing SARS-CoV S protein and stained with anti-His-APC. (c) Neutralization curves of nanobody N235, S43 (positive control) and R14 (negative control) against pseudotyped SARS-CoV in Vero cells in vitro. Experiments were independently repeated twice with similar results, and one representative curve is displayed. The IC_50_ values (μg/mL) from duplicates are presented as average.


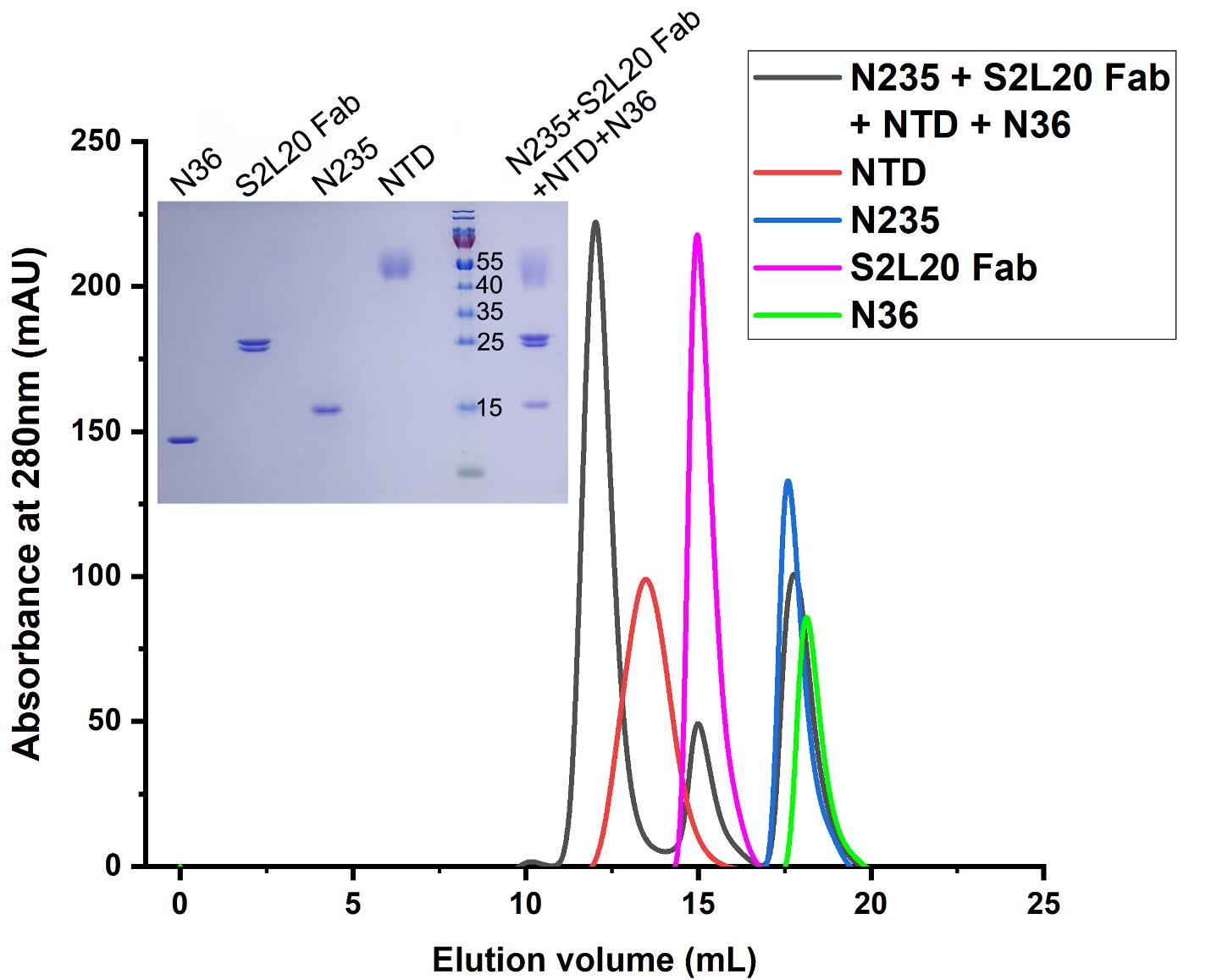


Figure. S4.

Gel filtration profile of SARS-CoV-2 BA.1 NTD and N235. The BA.1 NTD, N235, N36 and S2L20 Fab proteins elute as single monomer peaks in the gel filtration curves. The BA.1 NTD/N235/S2L20 complex displays a shifted complex peak. All the samples were assessed by SDS-PAGE.


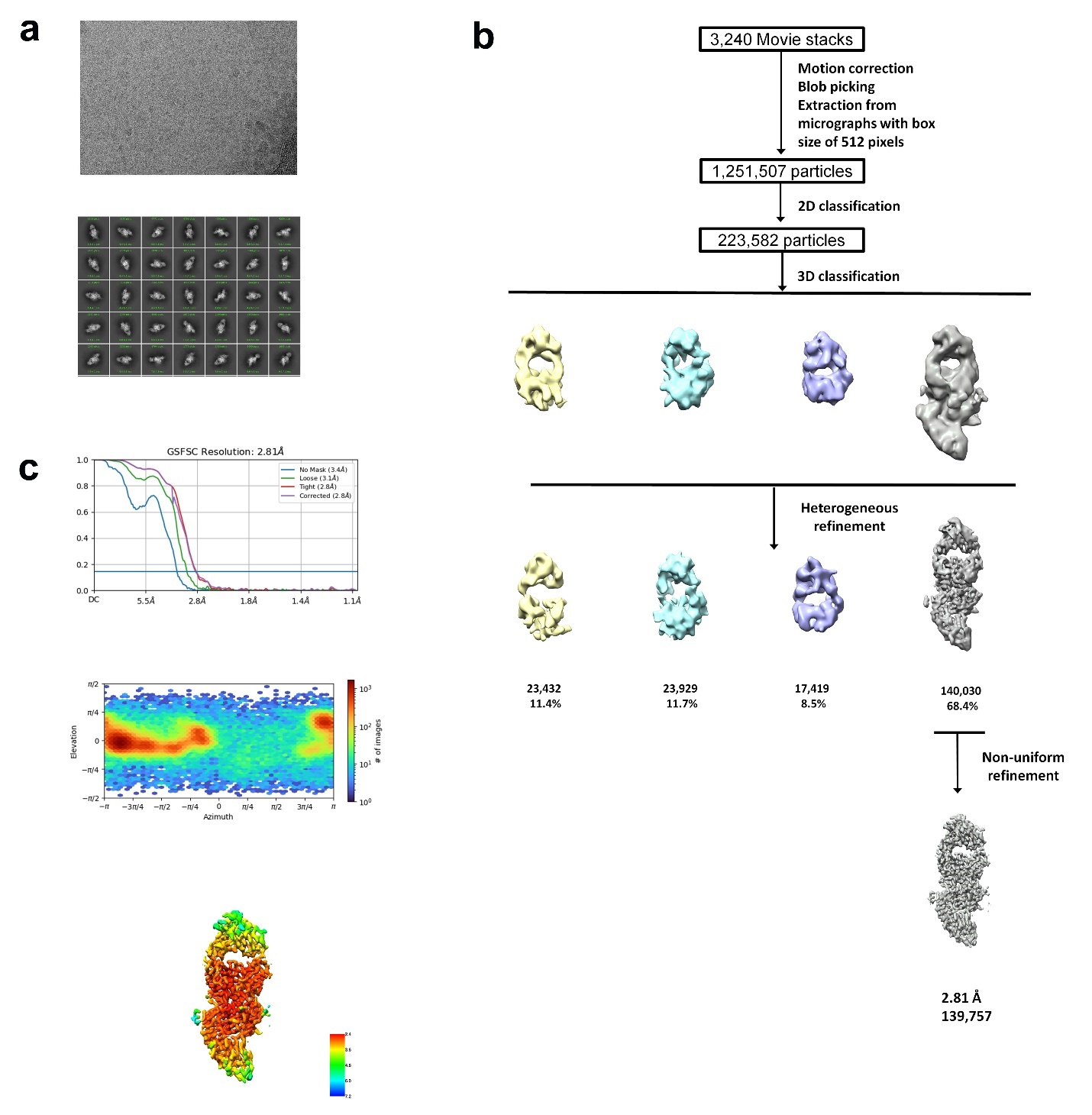


Figure. S5.

Cryo-EM data processing of N235/S2L20/BA.1 NTD complex. (a) Representative cryo-EM micrograph and 2D classes. (b) Workflow for the N235/S2L20/BA.1 NTD complex 3D reconstructions. (c) The gold-standard Fourier shell correlation (FSC) curves of the final EM map, the viewing direction distribution plot and the cryo-EM maps colored by local resolution for the N235/S2L20-Fab/BA.1-NTD complex.


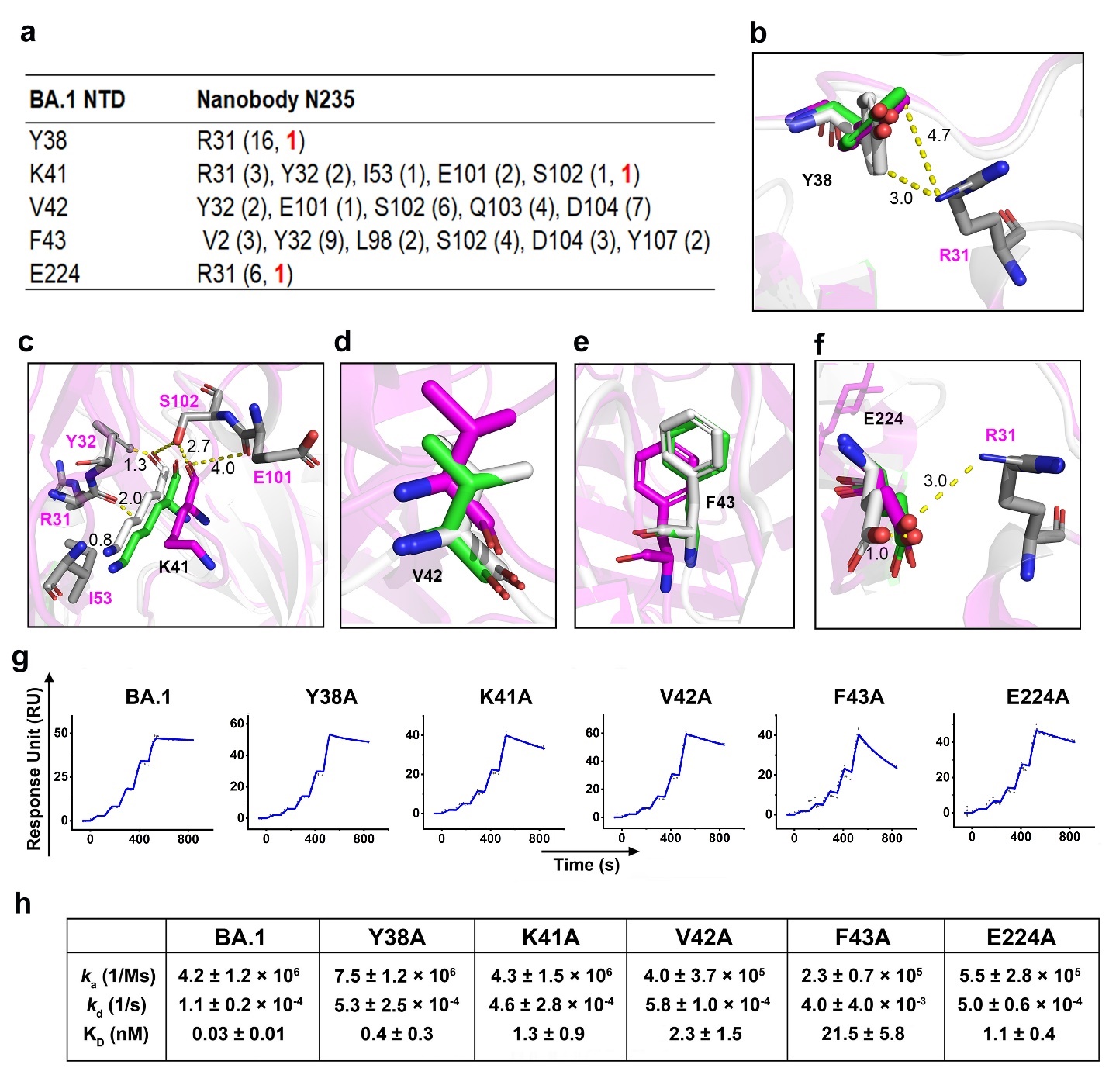


Figure. S6.

The epitope validation by point mutations of BA.1 NTD. (a) The tested epitope within BA.1 NTD. The alignment of amino acid Y38 (b), K41 (c), V42 (d), F43 (e) and E224 (f) within BA.1 NTDs from the structure BA.1 NTD (PDB: 7wvn, in white), S2L20/NTD (PDB: 7tlz, in green) and N235/S2L20/NTD (in this study, in magenta), respectively. (g) The binding kinetics of N235 to the NTD mutants obtained using a Biacore 8K system in single-cycle mode. (h) Kinetic and affinity values (*k*_a_, *k*_d_, and *K*_D_) are the mean ± standard deviation (s.d.) of three independent results. The absence of annotated *k*_d_ and *K*_D_ represents no detectable dissociation from the NTDs. Dashed line underneath represents the raw data, and the kinetic fit is shown as a solid line.


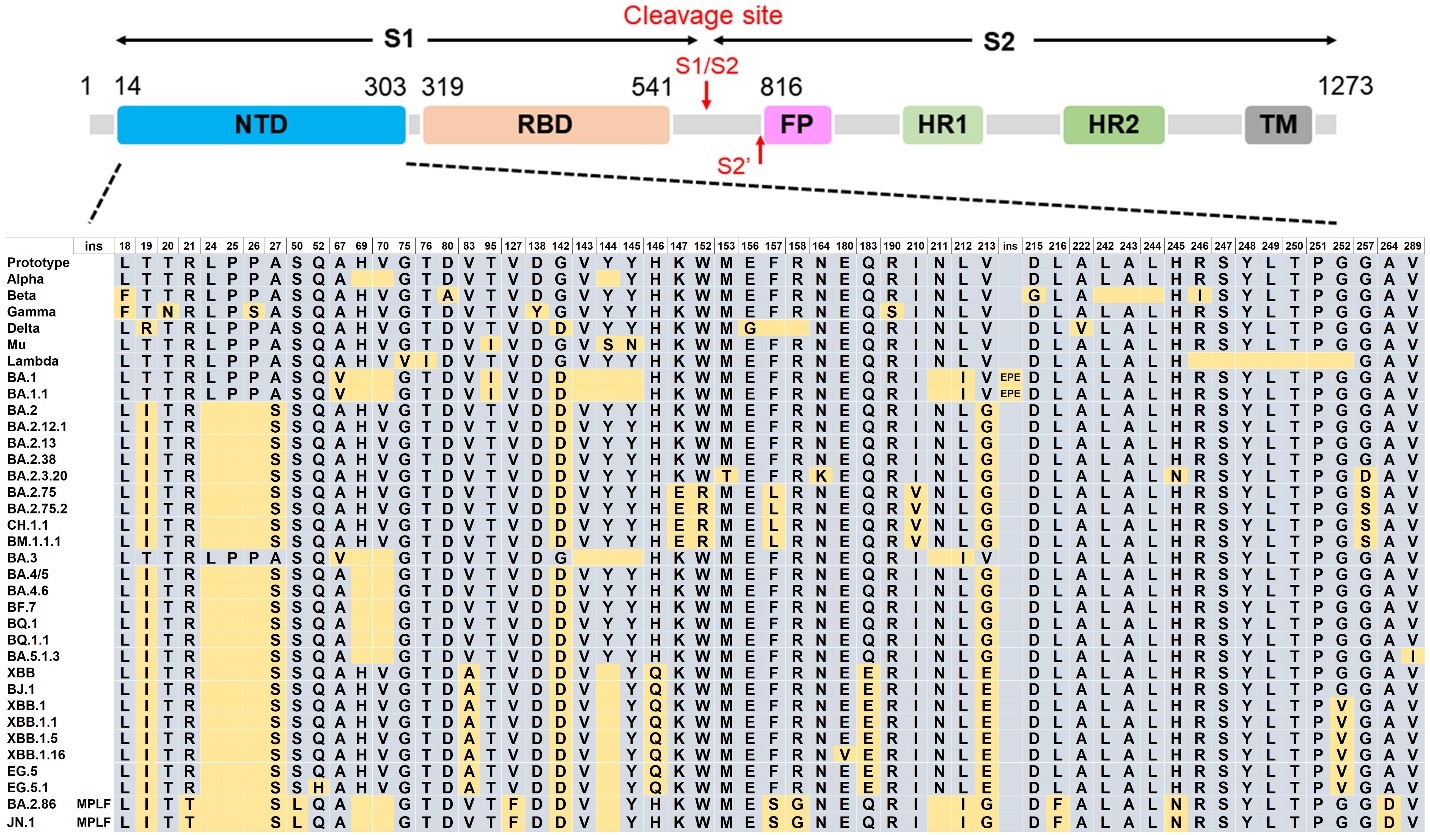


Figure. S7.

Overall topology of the SARS-CoV-2 S glycoprotein. NTD, N-terminal domain; RBD, receptor binding domain; FP, fusion peptide; HR, heptad repeat; TM, transmembrane region. The amino acid substitutions on NTDs of four previous VOCs and current Omicron sub-variants are displayed.


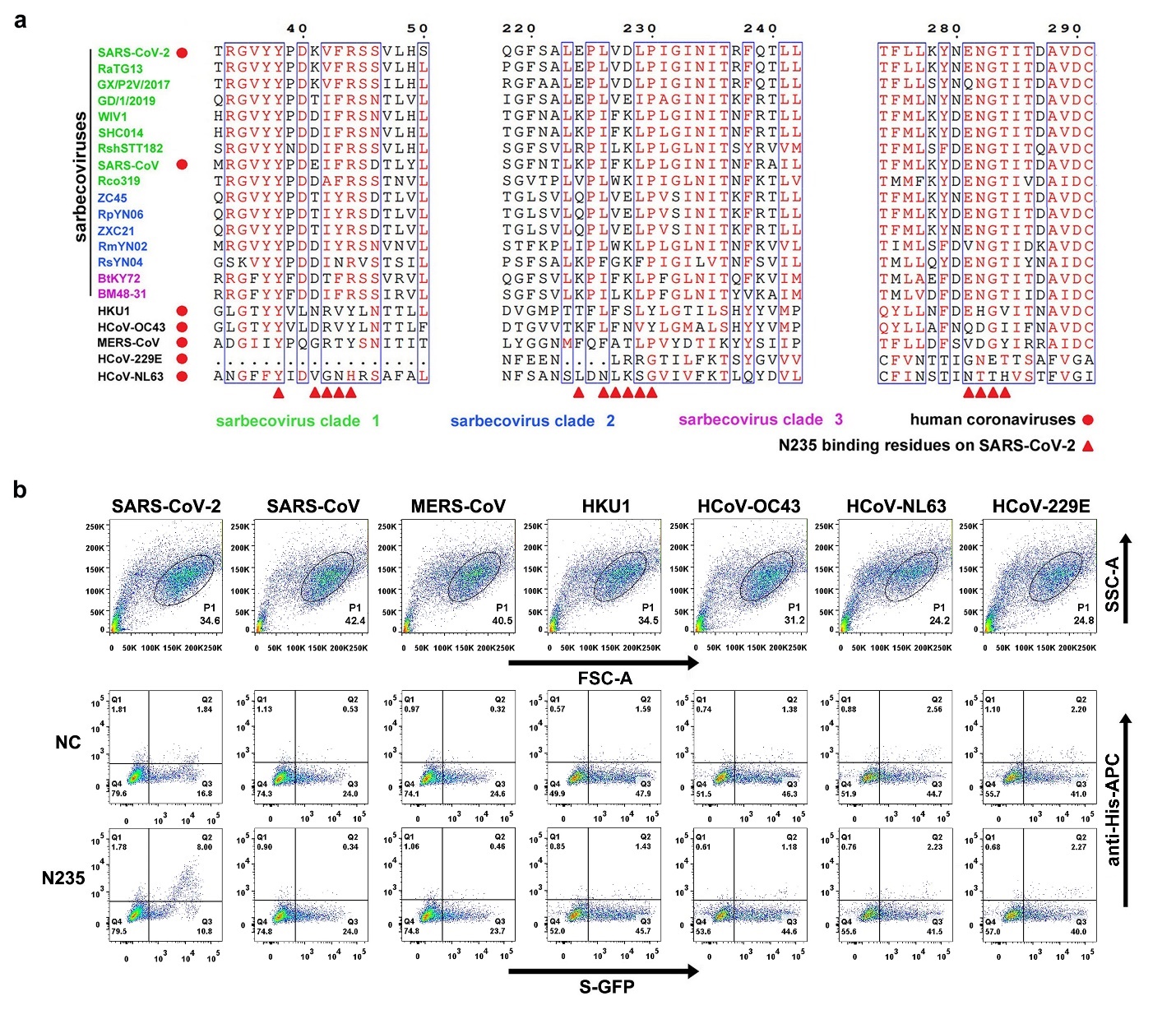


Figure. S8.

The cross-reactive of N235 against CoVs. (a) Sequence alignment of CoVs. Seven CoVs that were reported to infect humans are noted with red rounds. The binding residues on SARS-CoV-2 NTD are represented with red triangles. (b) FACS-based binding assay between N235 and seven human CoVs. GFP-fused S protein was transiently expressed on the surface of BHK-21 cells and stained with anti-His antibodies, which were pre-incubated with His-tagged N235. Experiments were performed twice, and one representative data is displayed. ‘NC’ presents negative control that was performed without nanobody protein. Cells were gated based on the FSC-A and SSC-A (P1). ‘SARS-CoV-2’ represents that PBS was incubated with BHK-21 cells expressing SARS-CoV-2 S protein and stained with anti-His-APC. ‘SARS-CoV’ represents that PBS was incubated with BHK-21 cells expressing SARS-CoV S protein and stained with anti-His-APC. ‘MERS-CoV’ represents that PBS was incubated with BHK-21 cells expressing MERS-CoV S protein and stained with anti-His-APC. ‘HKU1’ represents that PBS was incubated with BHK-21 cells expressing HKU1 S protein and stained with anti-His-APC. ‘HCoV-OC43’ represents that PBS was incubated with BHK-21 cells expressing HCoV-OC43 S protein and stained with anti-His-APC. ‘HCoV-NL63’ represents that PBS was incubated with BHK-21 cells expressing HCoV-NL63 S protein and stained with anti-His-APC. ‘HCoV-229E’ represents that PBS was incubated with BHK-21 cells expressing HCoV-229E S protein and stained with anti-His-APC.


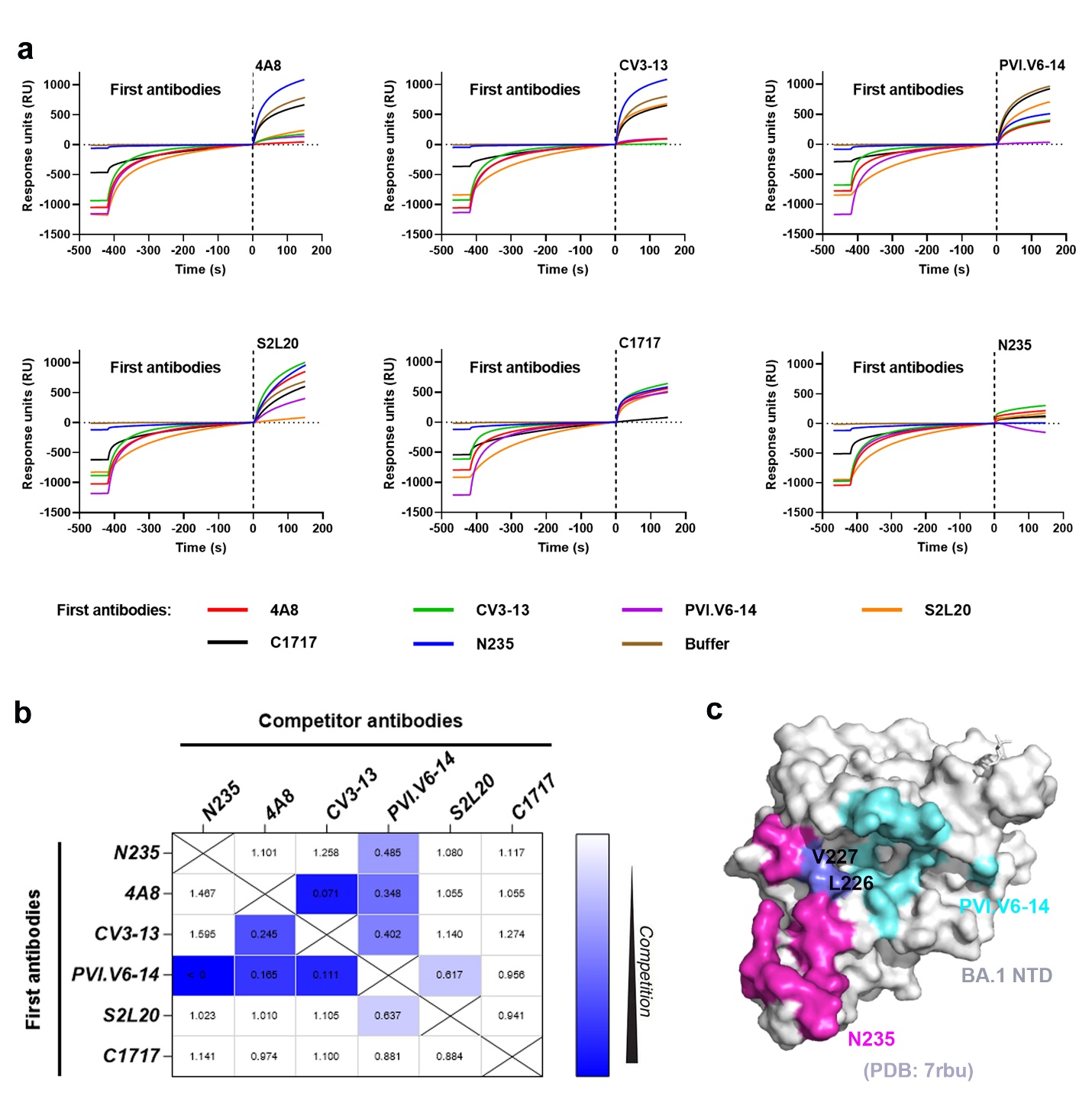


Figure. S9.

Competitive binding assay of NTD-binding antibodies found in this study (N235) and others (4A8, CV3-13, PVI.V6-14, S2L20 and C1717). (a) Competitive binding curves. The biotinylated SARS-CoV-2 PT NTD was immobilized on SA chips and then antibody flowed through the chip in the presence of first antibody on Biacore 8K system (GE Healthcare). One representative of two independent experiments is shown. (b) ‘Checkerboard competition’ is shown in heatmap. Numbers refer to the shift when the second antibody is added to the preformed antibody-NTD complex. One representative of two independent experiments is shown. (c) The footprint of N235 (light magenta) and PVI.V6-14 (aquamarine) mapped onto an NTD monomer (PDB: 7rbu). The overlapping residues L226 and V227 in the NTD region were shown in slate color.


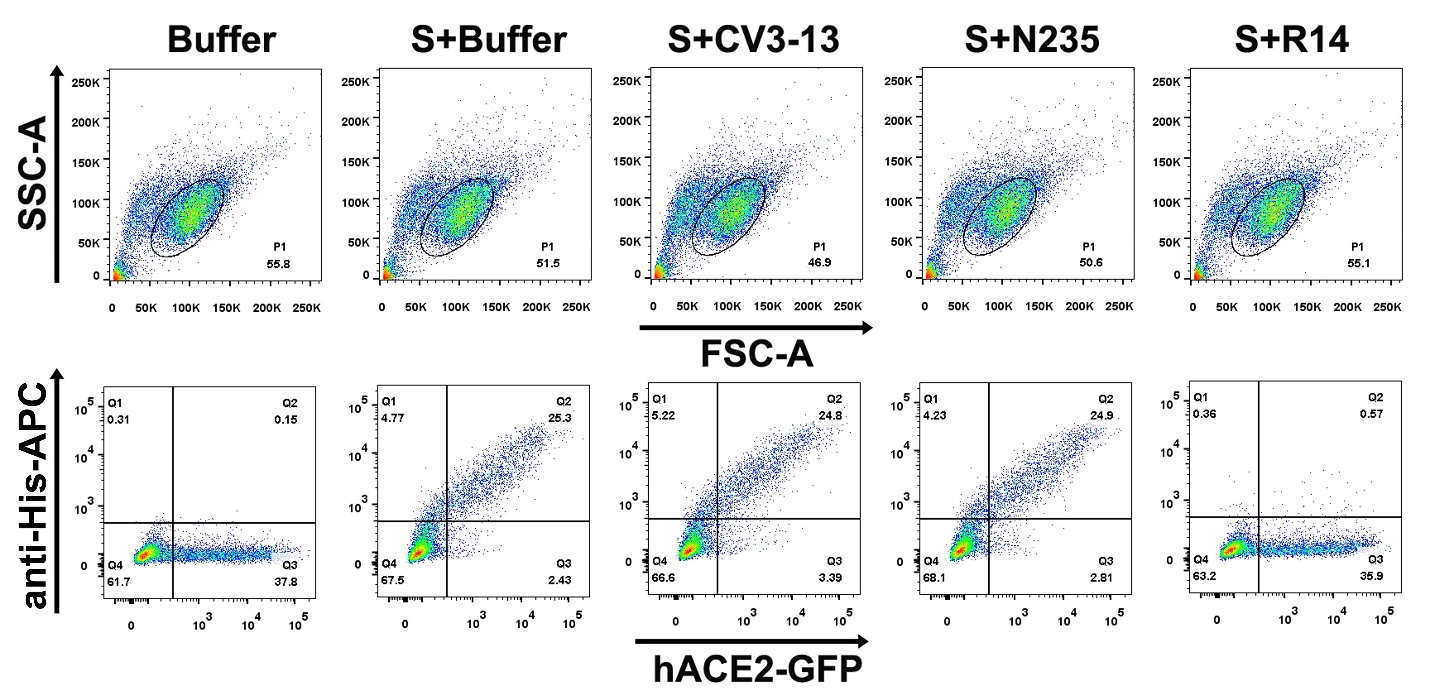


Figure. S10.

Gating strategy and blocking evaluation. FACS-based assay showed that N235 did not block SARS-CoV-2 S binding to human ACE2 (hACE2). hACE2-GFP fusion protein was transiently expressed on the surface of BHK-21 cells and stained with SARS-CoV-2 S proteins, which were pre-incubated with human Fc-tagged N235. Experiments were performed twice, and one representative data is displayed. Then flow cytometry was conducted using Fortessa. Cells were gated based on the FSC-A and SSC-A (P1). ‘Buffer’ represents that PBS was incubated with BHK-21 cells expressing ACE2-GFP protein and stained with anti-His-APC. ‘S+Buffer’ represents that BHK-21 cells expressing ACE2-GFP protein was incubated with SARS-CoV-2 S protein which were pre-incubated with PBS, and stained with anti-His-APC. ‘S+CV3-13’ represents that BHK-21 cells expressing ACE2-GFP protein was incubated with SARS-CoV-2 S protein which were pre-incubated with mAb CV3-13, and stained with anti-His-APC. ‘S+N235’ represents that BHK-21 cells expressing ACE2-GFP protein was incubated with SARS-CoV-2 S protein which were pre-incubated with human Fc-tagged N235, and stained with anti-His-APC. ‘S+R14’ represents that BHK-21 cells expressing ACE2-GFP protein was incubated with SARS-CoV-2 S protein which were pre-incubated with human Fc-tagged R14, and stained with anti-His-APC.


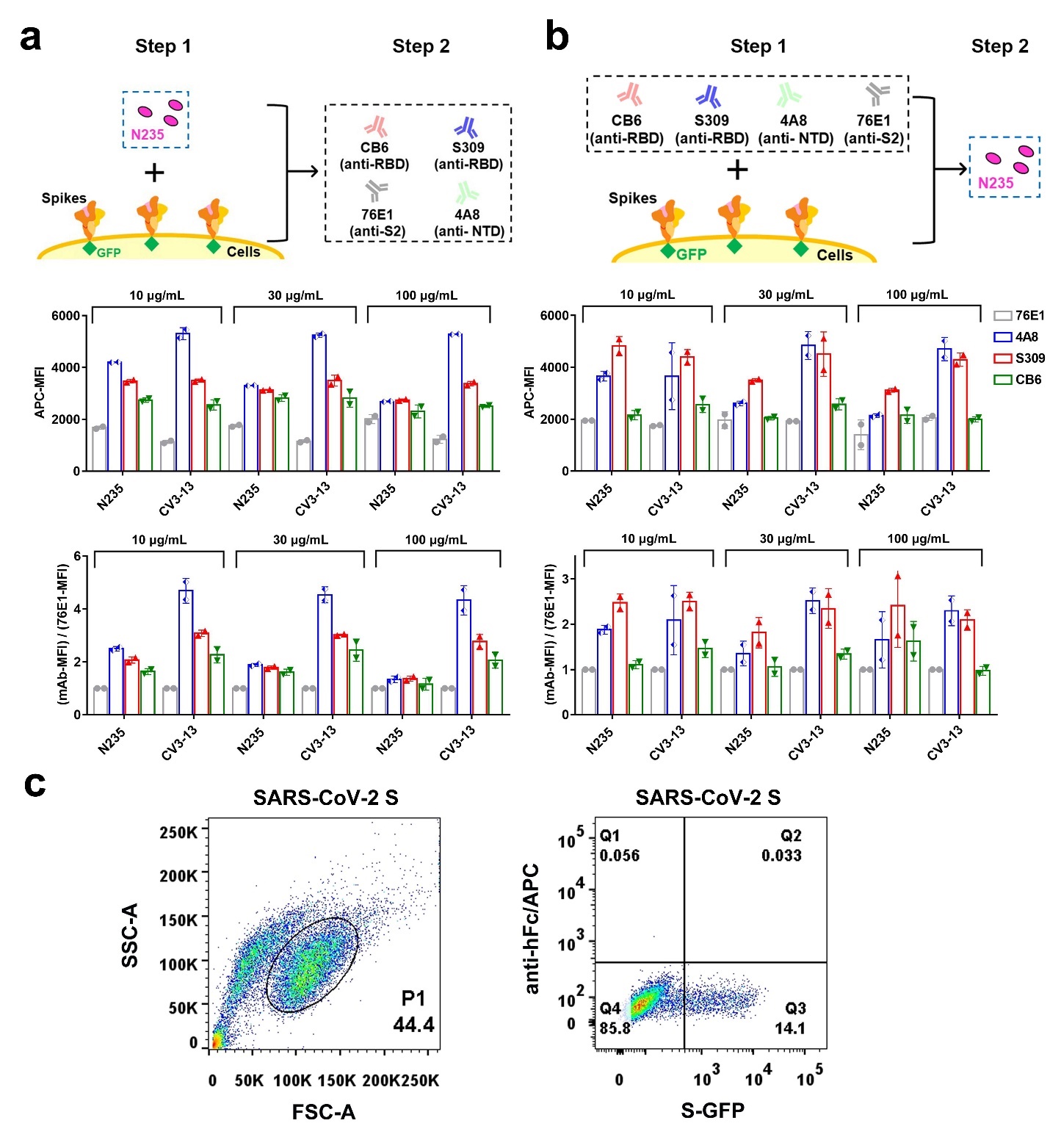


Figure. S11.

Comparison of mean fluorescence intensity (MFI). (a) The GFP-fused S proteins were transiently expressed on the surface of BHK-21 cells and stained with His-tagged N235 or CV3-13 Fab in 10, 30, and 100 μg/mL, respectively. The complex proteins were then incubated with RBD-targeting antibody CB6, S309, S2-targeting antibody 76E1, and NTD-targeting antibody 4A8. (b) The GFP-fused S proteins were transiently expressed on the surface of BHK-21 cells and stained with RBD-targeting antibody CB6, S309, 76E1, and 4A8 before incubation with His-tagged N235 or CV3-13 Fab in 10, 30, and 100 μg/mL, respectively. The first row illustrates the overview of flow cytometry assay process. In the second row, the vertical axis represents the APC-MFI of mAbs. In the third row, the vertical axis represents the level of S1 shedding, calculated by dividing the APC-MFI of cells with surface-expressed S treated with CB6, 4A8 and 76E1 by the APC-MFI of the 76E1-treated group. (c) Cells in Figure 4 and S11 were gated based on the FSC-A and SSC-A (P1) ‘SARS-CoV-2 S’ represents that BHK-21 cells expressing SARS-CoV-2 S protein and stained with anti-hFc/APC.


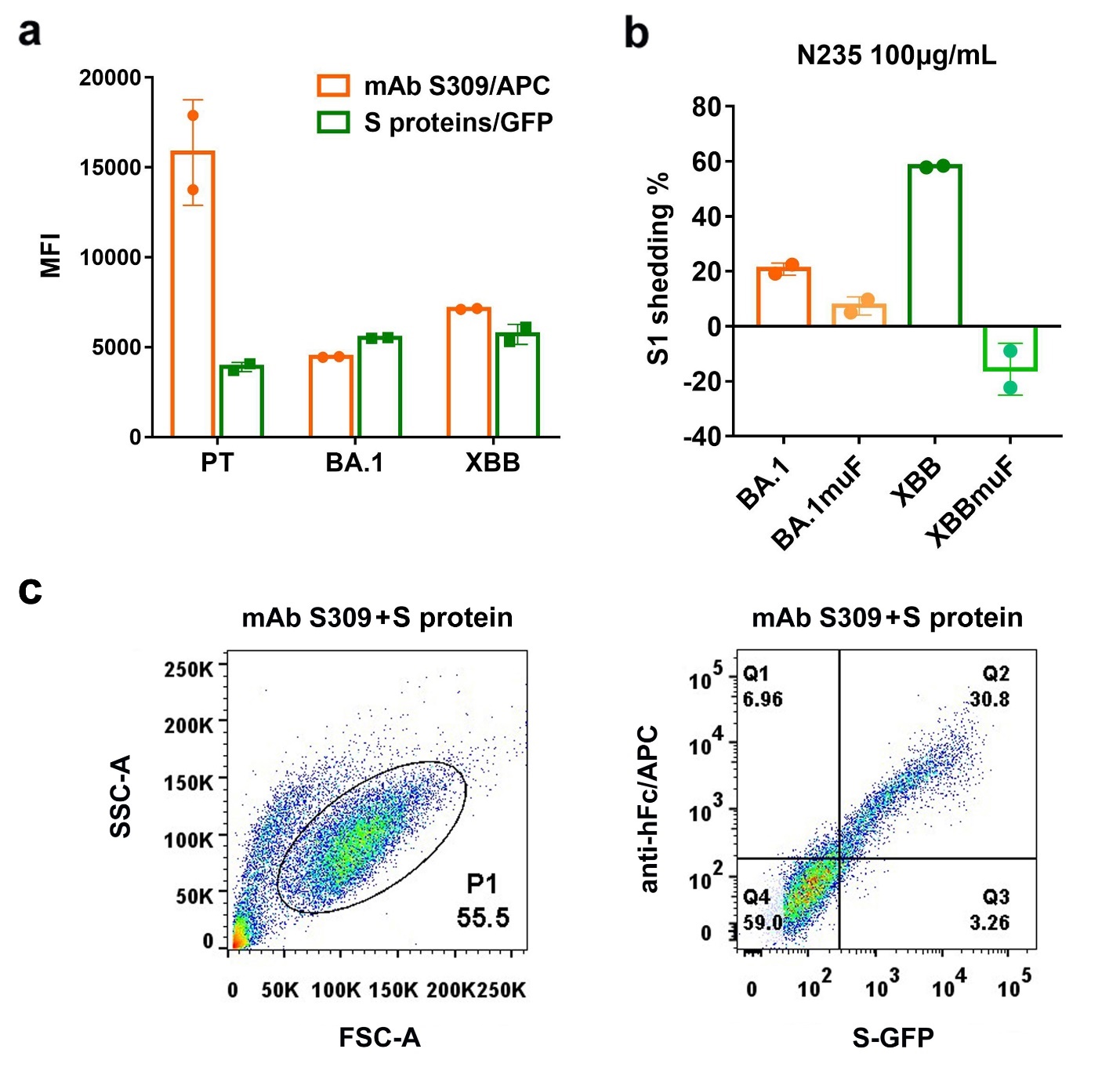


Figure. S12.

Comparison of mean fluorescence intensity (MFI). (a) The GFP-fused S proteins of prototype (PT), BA.1 and XBB were transiently expressed on the surface of BHK-21 cells and stained with mAb S309. Experiments were performed twice, and one representative is displayed. (b) The GFP-fused S proteins of BA.1 and XBB, as well as ones with inactive furin site (Δf) were transiently expressed on the surface of BHK-21 cells and incubated with His-tagged N235 in 100 μg/mL, followed by staining with mAb S309. Experiments were performed twice, and one representative is displayed. The percentage of S1 shedding is calculated by subtracting the MFI of cells with surface-expressed S treated with N235 from the MFI of the PBS-treated group, and then dividing it by the MFI of the PBS-treated group. (c) Cells in Figure S12 and S14 were gated based on the FSC-A and SSC-A (P1). ‘mAb S309+S protein’ represents that mAb S309 was incubated with BHK-21 cells expressing S protein and stained with anti-hFc/APC.


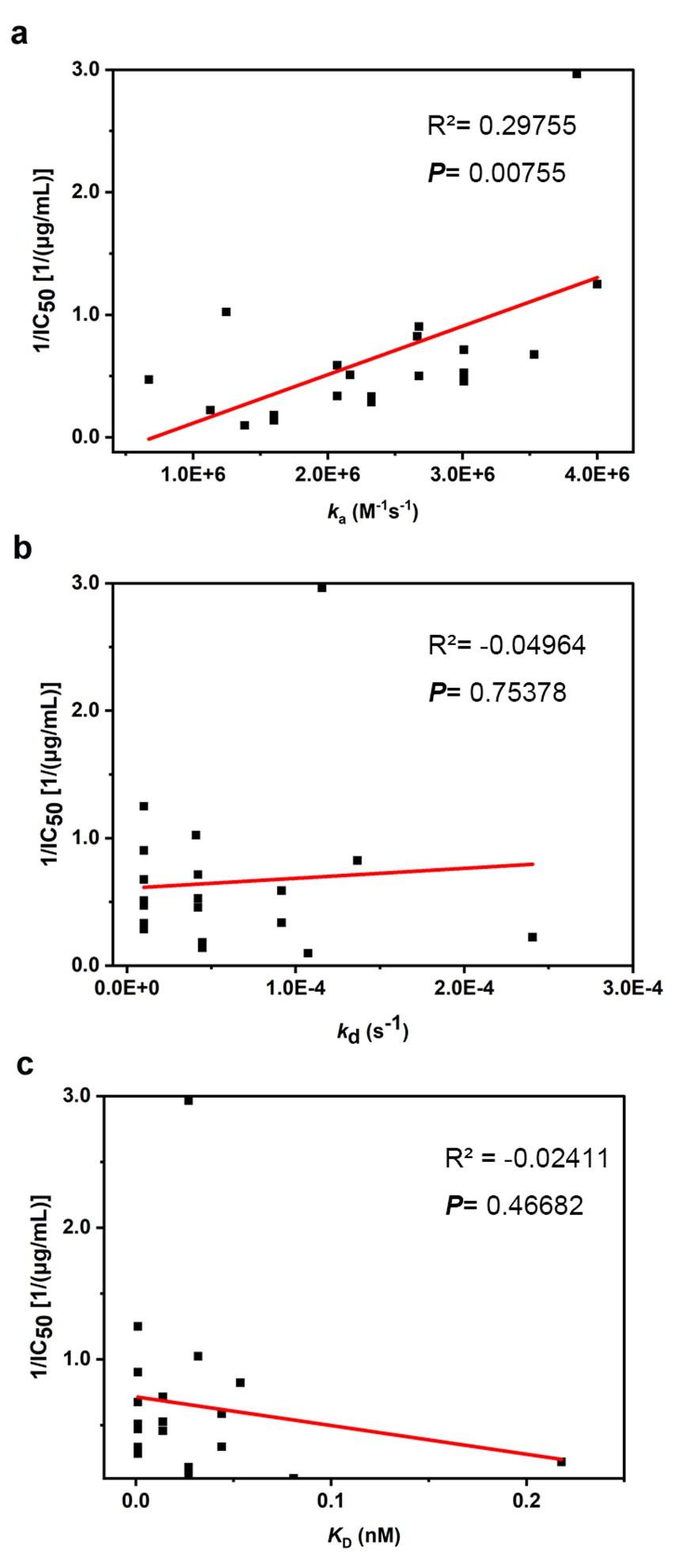


Figure. S13.

Correlation of binding strength and neutralization activity of N235. Reciprocal of *K*_D_ (nM) and 1/IC_50_ [1/(μg/mL)] for N235 against the tested variants are set as horizontal and vertical axes, respectively. The red line is the result of linear fit of the data points. When assessing the affinities of N235 with NTDs, where no detectable dissociation occurred, the *k*_d_ values were considered to be 10^-5^ s^-1^, and *K*_D_ values were considered to be 0.001 pM, based on the range of kinetic and affinity measurements. R^2^ and *P* value were indicated alongside.


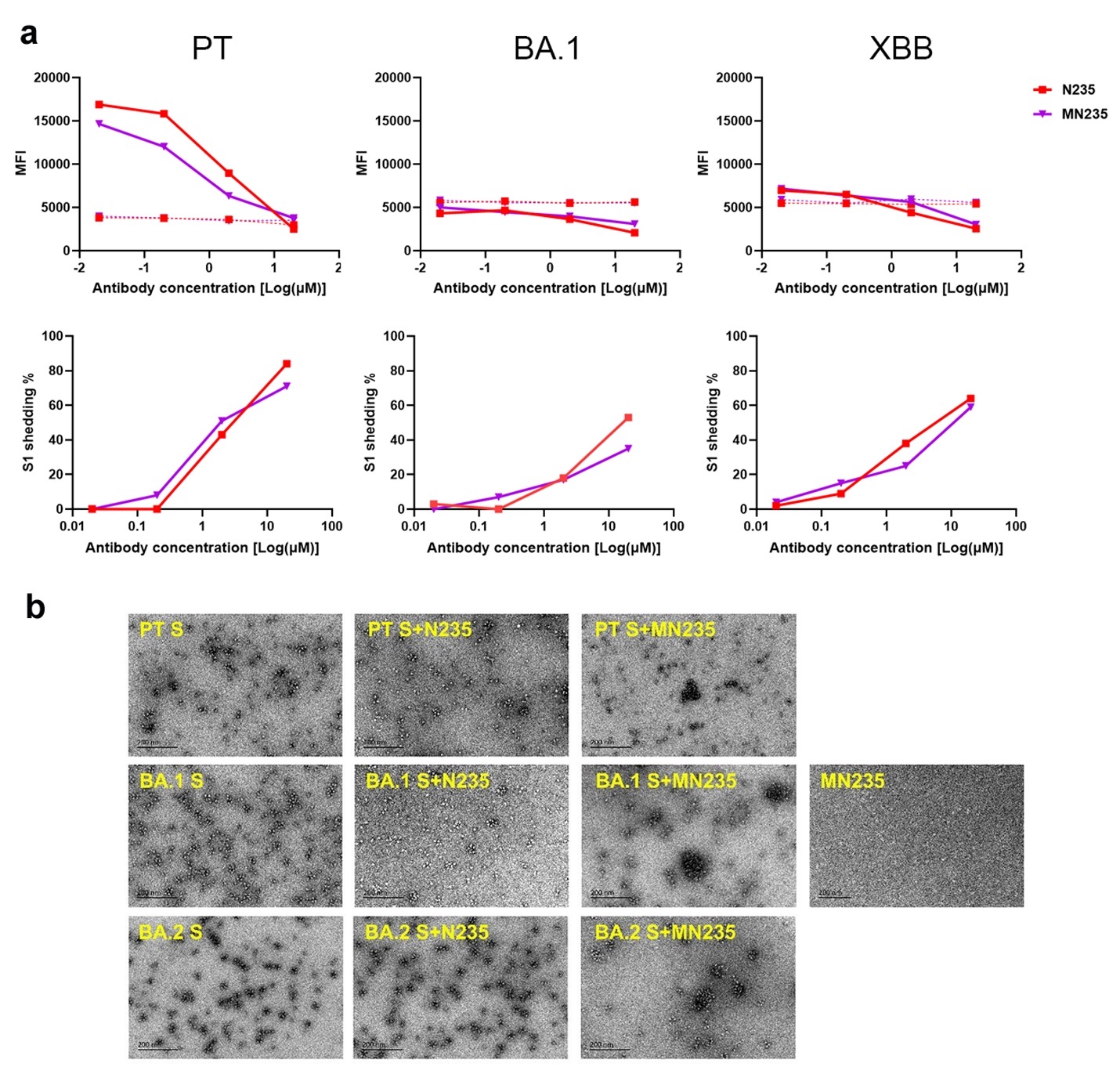


Figure. S14.

The neutralization mechanism of IgM-like MN235. (a) The flow cytometry-based assays to test S1 shedding by comparing mean fluorescence intensity (MFI). The GFP-fused S proteins of PT, BA.1 and XBB were transiently expressed on the surface of BHK-21 cells and stained with mAb S309 before His-tagged N235 or MN235 in dilution series, respectively. In the first row, MFI values represent the level of the rest S1 on cells. The dashed line represents the expression level of the S proteins indicated by GFP MFI values. In the second row, the percentage of S1 shedding is calculated by subtracting the MFI of cells with surface-expressed S treated with N235 or MN235 from the MFI of the PBS-treated group, and then dividing it by the MFI of the PBS-treated group. (b) Representative negatively stained images of protein particles. The morphology of protein particles was determined using TEM (JEM-1400). Experiments were independently repeated twice with similar results, and representative images are displayed.


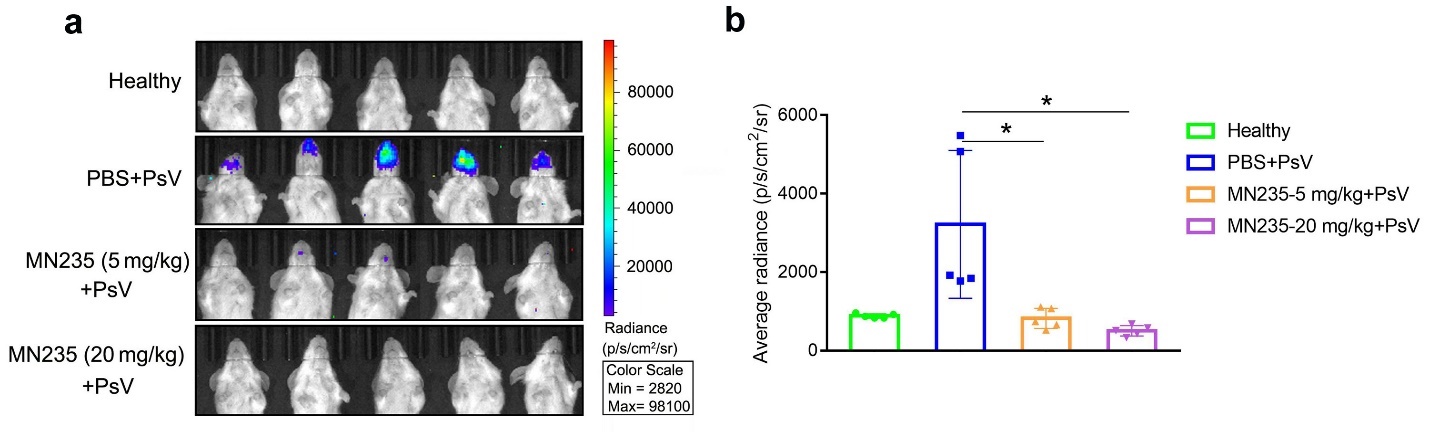


Figure. S15.

The prophylactic efficacy of MN235 against Omicron XBB pseudovirus *in vivo*. (a) 6-8-week-old female BALB/c mice were infected by i.n. with Ad5‑hACE2. Five days later, mice were challenged with luciferase-expressing SARS-CoV-2 Omicron XBB pseudovirus (n= 5 per group). Mice were imaged 24 h after pseudovirus infection. (b) The bioluminescence signal in the nasal passage was quantified. Data are presented as mean ± s.d. Student’s *t*-test was used to analyze differences between groups. **P* < 0.05.


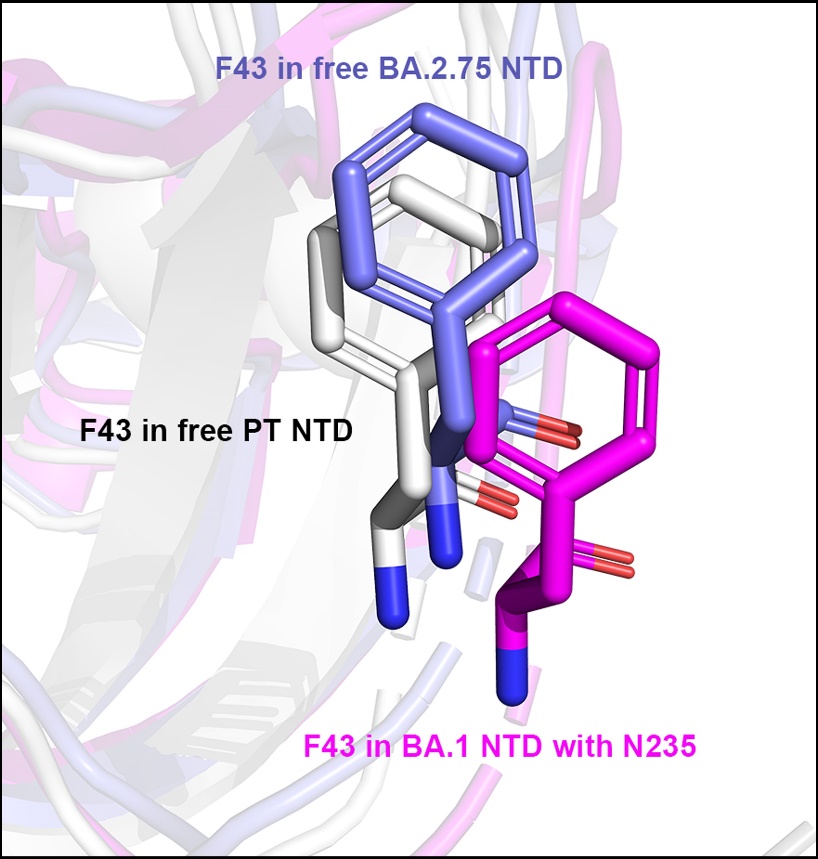


Figure. S16.

The alignment of amino acid F43 in the free PT NTD (PDB:7ddd, in white) with those in the free BA.2.75 NTD (PDB: 8gs6, in slate) and the BA.1 NTD (in this study, in magenta). A significant 1.5 Å shift in the main chain was observed for BA.2.75 NTD compared to PT NTD.

Table S1.

Cryo-EM data collection, refinement and validation statistics.

|  | N235/S2L20-Fab/BA.1-NTD  (PDB ID: D_1300039002) |
| --- | --- |
| **Data collection and processing** |  |
| Magnification | 130k |
| Voltage (kV) | 300 |
| Electron exposure (e–/Å^2^) | 60 |
| Defocus range (μm) | -1.0 ~ -2.0 |
| Pixel size (Å) | 0.54 |
| Symmetry imposed | C1 |
| Initial particle images (no.) | 223,582 |
| Final particle images (no.) | 139,757 |
| Map resolution (Å)  FSC threshold | 2.81  0.143 |
|  |  |
| **Refinement** |  |
| Initial model used (PDB code) | 7TM0, 6V80 |
| Model resolution range (Å) | Up to 3.50 |
| Map sharpening *B* factor (Å^2^) | 110 |
| Model composition  Non-hydrogen atoms  Protein residues  Ligands | 6071  769  60 |
| R.m.s. deviations  Bond lengths (Å)  Bond angles (°) | 0.004  0.946 |
| Validation  MolProbity score  Clashscore  Poor rotamers (%) | 1.62  5.62  0.00 |
| Ramachandran plot  Favored (%)  Allowed (%)  Disallowed (%) | 95.45  4.55  0.00 |

Table S2.

Nanobody N235 in complex with SARS-CoV-2 BA.1 NTD. The numbers in parentheses of nanobody N235 residues represent the numbers of vdw contacts the indicated residues conferred. The numbers behind comma suggest numbers of potential H-bonds between the pairs of residues. vdw contact was analyzed at a cutoff of 4.5 Å and H-bonds at a cut off of 3.5 Å.

| Nanobody N235 | SARS-CoV-2 BA.1 NTD |
| --- | --- |
| Q1 | E281 (10), N282 (2), NAG282 (4) |
| V2 | F43 (3) |
| G26 | N282 (13), NAG282 (5) |
| R31 | Y38 (16, **1**), K41 (3), E224 (6, **1**), P225 (1), G283 (1), T284 (2) |
| Y32 | K41 (2), V42 (2), F43 (9), G283 (3) |
| I53 | K41 (1) |
| P54 | L226 (2), D228 (5) |
| A55 | V227 (1), D228 (8), L229 (3), P230 (3) |
| L98 | F43 (2) |
| E101 | K41 (2), V42 (1) |
| S102 | K41 (10, **1**), V42 (6), F43 (4) |
| Q103 | V42 (4) |
| D104 | V42 (7), F43 (3), R44 (6) |
| Y107 | F43 (2) |
